# Spirit of the Foreign Periodicals, &c.

**Published:** 1837-01-01

**Authors:** 


### Spirit of the Foreign Periodicals, &c.

German Contributions to Practical Medicine. Communicated by Robert Thacker, M.D. Derby.

The following interesting observations on the diseases and state of medicine in Chili, are condensed from Professor Poeppig's "Travels in Peru, Chili, and on the River Amazon."*

"The population of Chili is rapidly on the increase, owing to the many advantages enjoyed by the inhabitants in their political position, and also to the circumstance that they are very little exposed to the diminishing influence of disease. Indeed, whatever has been said by earlier travellers of the remarkable healthfulness of the climate, may be credited in its fullest extent. Neither the extreme aridity of the northern provinces, nor the long-continuing and severe rains in the southern, would appear to exert any particular influence on the duration of life or on the health of the natives. In Mendoza and San Juan, which are only separated from Chili by the chain of the Andes, all classes of the inhabitants suffer severely from scrofula, and cretinism is not infrequent; in Chili, on the contrary, the latter is entirely unknown, and some traces of the former are only occasionally observed among the residents of the northern parts of the country at the foot of the Andes. The foreigner who makes a lengthened stay in Peru may regard himself as fortunate if he escape a tertian intermittent, which will frequently bid defiance to the most careful and judicious treatment. In Chili, on the other hand, there is no fear whatever of this disease, indeed, many Peruvians affected with it come yearly into Chili with the view of regaining their health. The regularity of the meteorological phenomena, the gradual manner in which changes of temperature take place, the purity of the water used for drinking, the constant winds, and the absence of marshes and damp woods, are the main causes to which the Chilians of all grades are indebted for the particularly good health they enjoy. The infectious diseases occasionally introduced soon lose their virulence under the sky of Chili. The contageous putrid fever of Guayaquils is only remembered to have once appeared at the commencement of the present century in some of the northern sea-ports. Small-pox alone is of frequent occurrence, and the fatality occasioned by it is sometimes considerable. The opinion is commonly entertained that this disease always comes from Peru, probably because it generally makes its first appearance at some seaport station. Epidemics of small-pox usually occur towards the end of the Winter, and from July to November, and it is observed that they assume a more malignant character as the weather becomes fine and warm, and the intervals of rain are longer. The mode of life of the lower classes, and the great want of cleanliness in their habitations, are circumstances favourable to the propagation of the disease; and under this head we must not omit to mention the deplorable maltreatment experienced by the sick, who are confined in the most secluded corner of the house, and dosed with all kinds of rubbish, large fires are lighted, all access of fresh air is carefully guarded against, and the poor patient almost smothered under woollen cloths—a proceeding, it may be remarked, which is observed in almost every case of illness. About 30 years ago, the Spanish government transmitted cow-pox matter to Chili by a man-of-war, but the indolence of the inhabitants has hitherto formed a serious obstacle to the introduction of a

* This excellent work was published not long ago, and is the subject of a highly laudatory article in a late number of the "Foreign Quarterly Review." The author is a talented, observant, and erudite man.

general practice of vaccination. Companies, supported by the government, are now instituted; their object is to extend vaccination, and this benefit is conferred on the poor gratuitously. At 8 localities, a vaccinating physician has been stationed, with a yearly salary of from 240 to 480 duces, and one of these gentlemen, named Alvear, fortunately discovered the vaccine pustule in the cows of Chili. In the years 1828, 1831 and 32, an epidemic of a peculiarly malignant character prevailed, and the people, driven by fear, came in great crowds to be vaccinated, so that from 1830 to 1832, the total number of those vaccinated amounted to 37,834. The Indians suffer far more from the ravages of small-pox than the Chilians.

Acute diseases seem on the whole to occur but seldom, with the one exception of typhus, which here, however, is not wont to destroy the powers of life so suddenly as in other countries. In the towns and cities the mortality is greater than in the country. With respect to chronic affections, the secondary symptoms of syphilis are of the most frequent occurrence; in fact, lues venerea may be considered epidemical in Chili; yet such is the beneficial influence of the climate, that in this country, as indeed throughout the whole of South America, this disease, formidable as it is in northern Europe, is almost entirely divested of its terrors. The countryman pays attention to his diet, drinks infusion of sarsaparilla, and not infrequently recovers completely without the use of metallic remedies. In large towns, however, the disease is not so easily removed, which is partly to be ascribed to the empirical treatment adopted; for here, as in all other countries, how remote soever, of the extra-European world, LEROY's celebrated panacea plays a very prominent part, and many other nostrums of Spanish origin are also high in favour. Among the Indians of Chili, lues is not common, and it does not appear to have been ever communicated to the races on the eastern side of the Andes.

It would seem that with the spread of civilization in Chili, diseases heretofore unknown, and which we may feel little disposed to regard as a consequence of our social condition, were at the same time introduced. Thus it is a fact that scarlet fever was not known up to 1831, when it suddenly broke out as a violent epidemic. Yet it was mild, for according to the report of the protomedicus, among 547 cases, there were only 25 exhibiting a malignant type (under the form of Febr. scarlat. miliaris), in which typhoid symptoms supervened. Of the latter 6 or 7 died, but of the whole number only a twelfth part terminated fatally.

The science of medicine has hitherto made but small advances in Chili, and it is only within a very recent period that really competent men have begun to enjoy some portion of public esteem. Twenty years ago medicine was practised by men of colour and the most ignorant quacks, the hospitals were entirely under the control of the monks, and the few well-informed physicians, those, namely, who had received an European education, found in Lima a more lucrative field for their exertions than was offered to them any where in Chili. This state of things, however, no longer obtains, for the number of physicians is now even greater than necessity requires, and there is a college at Santiago at which medicine is taught, although on a somewhat compendious plan. Still there is a scarcity of educated practitioners in the country, where the healing art is chiefly exercised by matrons and old Indian women. The country people are tolerably familiar with the virtues of the rather numerous indigenous plants, but to many of these properties are attributed which the individuals in question do not really possess, and many other remedies, supposed to be of great efficacy, are solely indebted for the estimation in which they are held to a credulous superstition. When any particular part of the human body is the seat of disease, the remedy is sought for in the corresponding part of some animal, variously prepared; thus, for ophthalmic disorders, the carbonized eyes of birds of prey are administered, the claws of the condor are given for gouty pains of the hands, and the flesh of the smooth-skinned lizard for scurfy affections of the skin. For the treatment of ordinary cases, the simple materia medica of the country suffices."—*Poeppig's Reise in Chile, Peru und aufdem Amazonenstrome.* S. 524—531.

The following case of poisoning with Muriate of Baryta is condensed from Henke's Journal of Legal Medicine. We have deemed it of some importance as exemplifying the action of that salt on the human subject in poisonous doses. Professor Christison observes that little is known on this subject, and that no account has yet been published of the morbid appearances as they occur in man.

*Rare Case of fatal Poisoning with Muriate of Baryta. Communicated by Dr. Wach of Merseburg.*

On the morning of the 4th of December, 1835, Charlotte M. aged 42, the wife of a manufacturing chemist residing in the country, in the absence of her master took half an ounce of the powder from a paper which she believed contained Glauber's salt, and having dissolved it in warm water, swallowed the whole at once. Soon after she was seized with nausea, retching, twitching of the facial muscles, and convulsive snatching of the hands and feet, to which succeeded a violent vomiting of a muco-aqueous fluid, which the servant in attendance threw away. These symptoms continued with increasing severity, the twitching of the face and of all the limbs grew rapidly worse, and, before the nearest physician had arrived, she expired under the most violent convulsions, scarcely two hours from the time of taking the salt.

*Sectio cadaveris.*—The body was rather fat, the mouth firmly closed, the features were distorted, and the fingers spasmodically contracted — abdomen and præcordia sunk. In the cavity of the abdomen the great and the small omentum unusually red, their vessels being filled with blood, the stomach contracted, and the vasa brevia turgid with blood—the peritoneal coat of the stomach of a dark brown colour and much inflamed. At the distance of 3½ inches (Parisian measure), from the cardiac orifice, and 9 lines from the smaller curvature on the posterior wall, there was a perforation of the coats of the stomach of an oval form, and measuring three lines in diameter on the external surface, and 7½ on the inner. The edges were very much swollen, and the mucous membrane for the space of two inches around was thickened and covered, not with pus, but a bloody mucus. The entire mucous membrane of the stomach was highly inflamed, and covered with mucus and coagulated blood. The muscular coat, with the exception of the place where the perforation existed, was no where softened or attenuated, but in a normal state. The cardia and pylorus, the duodenum, jejunum, and ilium, were all in a high state of inflammation, the mucous membrane softened, thickened, and smeared with a bloody mucus. The small intestines contained several ounces of a brownish-red slimy fluid, mingled with clotted blood. The colon throughout its entire length was morbidly contracted, so that its caliber was even one-third less than that of the small intestine. Many broad ecchymosed patches, from half an inch to an inch in length, were observed on its inner surface. The pharynx and œsophagus were slightly inflamed. The convex surface of the liver adhered throughout to the diaphragm, and the blood flowing from incisions in this organ and in the spleen was thick and of a very dark colour. In the gall-bladder was a pale-yellow gall-stone of the size of a hazel-nut; the gall itself pale-yellow and watery. In the uterus were many little clots of coagulated blood, the bloodvessels of the ovaries turgid, the external and internal parts of generation in the virginal state. The lungs and brain were congested with thick black blood.

It may be remarked that the fatal powder was fully proved to be muriate of baryta. Dr. Wach observes that the adhesions between the liver and diaphragm, the gall-stone, the morbid condition of the gall, and the contracted state of the large intestine, in all probability existed at the time the poison was taken, and that the appearances in the uterus and ovaries might be dependent on the menstrual discharge; but, he adds, all the other morbid changes, including the perforation of the stomach, were certainly occasioned by the irritant action of the poison.—*Henke's Zeitschrift für die Staatsarzneikunde. Fünfzehnter Jahrgang.*—1835.

In the 15th and last volume of Siebold's Journal of Midwifery, is a communication illustrative of obstetric medicine in Denmark, by Dr. Neuerman, the same from whose paper in a late number of Rust's Magazine, and which, it will be remembered, was also compiled from Danish sources, we formerly made rather copious extracts, which, under the title, "Scandinavian Contributions to Practical Medicine," appeared in the 47th number of this Review. For the cases which follow, we are again indebted to Dr. Neuerman; they are selected from his communication in Siebold's Journal, and will be admitted, we think, to posses no ordinary interest, exemplifying, as they do, that rare deviation from nature, extra-uterine pregnancy. The last case, indeed, was probably not one of this nature, though bearing much resemblance in its extraordinary course and termination to several recorded instances of extra-uterine pregnancy.

*Case of Tubal Pregnancy, observed by Dr. Drejer of Copenhagen, Physician to his Majesty.*

A mason's wife, aged 33, the mother of five children, and who believed herself to be in the 5th month of another pregnancy, having distinctly felt the movements of the child, though for a fortnight they had altogether ceased,—was suddenly seized, after a breakfast consisting of tea and biscuit, with a burning pain in the abdomen, speedily followed by rigors, vomiting, and great prostration of strength. A physician residing in the neighbourhood prescribed a julep to allay the vomiting, but being himself indisposed, he did not see her again. In the afternoon she was visited by Dr. Drejer, who found her in a state of syncope and with every sign of anæmia, although no blood had passed from her. On recovering consciousness, she complained of agonizing pain in the abdomen and of retention of urine. There was no distention of the abdomen, and on examination the uterus was ascertained to be in its normal situation with the os uteri somewhat open, whence Dr. Drejer inferred that his patient was mistaken in supposing herself pregnant, and, attributing her sufferings to spasms, he directed the julep to be continued with the addition of a little musk and liq. anodyn. She died quietly an hour afterwards.

*Sectio Cadaveris.*—On pressing upon the abdomen, a hard rounded body was felt in the hypogastric region. Within the abdomen was observed, first, a large quantity of extravasated blood; this being removed, the placenta was discovered; it presented the appearance of a convex fleshy body, and was inserted into the middle of the right fallopian tube, which was bursted; a fœtus of the female sex, weighing 6½ ozs., and attached to the placenta by the umbilical cord, lay among the intestines. The placenta was 10½ inches in circumference. It was remarkable that the broad ligament and fallopian tube of the right side did not pass off from the uterus as they normally do, but proceeded from that part of the organ immediately adjoining the neck. It was not possible to pass a probe through either tube. The uterus, which lay in its natural position, was somewhat enlarged, the cervix filled with a white gelatinous substance, the os tincæ open, and the uterine cavity rather dilated and lined by a thick pale membrane. A corpus luteum was found in the left ovarium, but with the most minute examination no traces of one could be discovered in the right, a circumstance sufficiently perplexing, for as every conception necessarily produces a corpus luteum, and as the one found was of the size and appearance usual in the

5th month of pregnancy, it is not easy to conceive how the ovum could have got from the left ovarium into the right fallopian tube. It may be remarked that the patient in this case was subject to hysteria, and for some time had suffered from a pulmonary affection, but during this pregnancy, she had not complained of repeated attacks of pain in the belly, or of an impossibility of lying on the right side.

*Case of Extra-uterine Pregnancy, which occasioned a complete retroversion of the Uterus, and did not terminate fatally. By Professor Drejer. Communicated to the Royal Medical Society of Copenhagen, on the 21st of February,* 1833.

A woman, aged 36, of a thin and slender make, who in her 19th year had given birth to twins, and subsequently to two other children, the last of which was born 13 years before, was seized in June, 1831, after having passed one menstrual period without the occurrence of the usual discharge, with a violent spasmodic attack, attended with loss of consciousness. Antispasmodic medicines were administered, under the use of which she soon recovered. After the lapse of several months, during which the catamenia still continued absent, the epileptic paroxysm recurred, for which she was again successfully treated with antispasmodics. At this period the abdomen was tumid, and the breasts enlarged, and she had no doubt that she was in the fourth month of pregnancy. Her health continued tolerably good until the 25th of November, when she applied to Dr. Drejer on account of crampy pains in the belly and thighs. She was feverish, and had quite the appearance of having attained the 7th month of pregnancy; the breasts contained milk, and the movements of the child were perceptible. The symptoms of which she complained were relieved by pulvis refrigerans conjoined with musk, but some days afterwards suppression of urine came on, attended with straining and violent pain, and it was found exceedingly difficult to introduce the catheter, the orifice of the urethra being completely behind the pubis (a circumstance, as Dr. Neuerman remarks, which is regarded by Mende as a characteristic sign of retroversion of the uterus). By holding the instrument in a direction nearly perpendicular, it was at length passed into the bladder, much dark-coloured urine was drawn off, and alleviation of the symptoms followed. On examination through the vagina and rectum, the uterus was found to be completely retroverted; the os tincæ, raised above the pubis, could scarcely be reached by the finger, and a tumor was felt occupying the posterior part of the pelvic cavity; it had somewhat compressed the rectum, and yielded little to pressure in consequence of a superincumbent weight. Dr. Drejer was at first inclined to believe that his patient was not pregnant, but was convinced of the contrary by distinctly feeling the movements of the child through the abdominal parietes, and as it was clear that the tumor in the pelvis was the fundus uteri, and that it was impossible the os tincæ could occupy such a situation at the period of gestation to which this woman had arrived, he inferred that the case was one of extra-uterine pregnancy.*

The urine was drawn off twice a day, the accumulation of fæces obviated by the use of Glauber's salt, and, at the end of 14 days, the fundus uteri had evidently ascended towards the sacral promontory, and the os tincæ was somewhat lower; the excretions also were passed without assistance, and in the course of another fortnight, the uterus had nearly resumed its normal position. Gradually the movements of the child ceased. In January, 1832,

* Dr. Drejer does not seem to be aware of the fact that the uterus may remain in a certain degree of mal-position, with the os uteri still directed to, or raised above the pubis, even to the end of gestation. A case related by Dr. Merriman in the Med. and Phys. Jour. Vol. XVI. page 388, would convince him of this. We do not, however, impugn the accuracy of his diagnosis.—Tr.

she had painful bearing-down efforts, accompanied by uterine hæmorrhage, but these symptoms vanished in a few days, and she at length so far regained her health as to be able to leave her bed, complaining only of weakness. In February, at the period when she had calculated on being confined, violent hæmorrhage and bearing-down pains again came on, but they ceased as before in the course of a few days, after which the distention of the belly and breasts diminished. The hard tumor, which still remained in the abdomen, extended from the symphysis pubis to beneath the false ribs on the right side, where Dr. Drejer believed he could feel the breech of the fœtus, the head appearing to lie below, and the back along the right side and anteriorly; a position which led to the supposition that the fœtus was sacculated in the abdomen, and not contained either in the right ovary or its fallopian tube. In April the catamenia appeared, and afterwards continued to recur at regular intervals; the woman is now in good health, attends to her business, makes long journeys on foot, and is not incommoded by the tumor farther than that it is occasionally the seat of shooting pains, particularly during the menstrual periods. She is also subject to hysteria. She was made acquainted with her situation, and assured that she might, notwithstanding, reach an advanced age.

*Rare Case of Pregnancy, in which the Fœtus remained between two and three years in the Abdomen, and was at length extracted by an Operation. By Kjär, District Surgeon at Holstebroe, in Jutland. Read before the Royal Medical Society of Copenhagen, in* 1833.

In the Summer of 1827, a woman ætat. 39, in her fifth pregnancy, experienced violent pains in the abdomen whenever the child moved, and the fear of approaching abortion induced her on several occasions to call in a midwife, who after examination declared her to be not pregnant. About the middle of December, when she had calculated that her confinement would take place, the ignorant midwife again declared that no pregnancy existed, although the movements of the child could be seen and felt, and affirming that the good woman was certainly bewitched, she finally left her to her fate.

For several days the violent movements of the child were still felt, after which they ceased altogether. The poor woman lay in the greatest torment, shrieking with pain, and it was necessary to hold her in bed. The abdomen was excessively distended, and so sensitive, that even the weight of the bed-clothes was unbearable. It was 8 weeks after this that she was examined by a physician, who brought away a fleshy mass which was considered to be a portion of the placenta: nothing more was done. In the course of 5 weeks she was able to leave her bed; in the Spring she could walk a little out of the house, and a quarter of a year after the cessation of the movements of the child, the catamenia appeared: she, however, felt weak and poorly throughout the whole of the following Summer and Winter. About the latter end of the succeeding Spring, a tumor formed below the umbilicus, and gradually attained the size of a goose's egg; it was opened by a pitch plaster in June 1829, and discharged a large quantity of an inodorous fluid. At this time, as the writer afterwards learned, a piece of flesh closed the opening and was taken away, upon which was observed something resembling a navel-string. The belly collapsed, but again became distended, and the opening healed up. In 6 weeks it again broke out, and gave issue to thick and highly offensive fluid, the discharge of which was not followed by a diminution of the abdominal distention. On the 26th of September, the patient was visited by the writer, who found her in bed in a state of exhaustion, with a quick full pulse and dry tongue, the bowels confined, and micturition difficult, the wound was painful, and she complained of nausea, which occasionally amounted to vomiting, in consequence of the excessively offensive smell of the discharge.

The abdomen was hard, sensitive, and uniformly distended, the opening below the umbilicus large enough to admit a sixpence (zweigroschenstück), and protruding through it, a macerated mass covered with hair. Herr Kjär having removed this substance with the scissars, felt within the opening a bone with a jagged edge, and on withdrawing his finger, a large quantity of a thick putrid fluid escaped, and then a mass resembling rancid oil. Nothing unusual was discovered by an examination per vaginam; the cervix uteri, indeed, was rather long, but not at all dilated, and the os tincæ sufficiently open to admit with facility a probe, which, however, could not be introduced into the cavity of the uterus. Herr Kjär resolved to dilate the opening in the abdomen, and the operation was performed on the 4th of October, the patient having been previously removed for the purpose into the town infirmary. At this time she was greatly debilitated. The hands and feet were œdematous, and a successful issue seemed extremely doubtful.

The patient being placed upon a bed, the operator dilated the opening in the direction of the linea alba to the extent of three inches, resting the scalpel first upon a director, and then upon his finger; the skull of the fœtus, previously opened with Smellie's perforator, was now extracted, and at length, with the assistance of this instrument and of the scalpel, the rest of the skeleton was removed. There were no traces of the after-birth or navel-string, with the exception of a piece of the latter, an inch and a half in length, which was attached to the umbilicus. The brain and intestines had also totally disappeared. The sac in which the fœtus was contained, was everywhere adherent to the surrounding structures; its parietes presented several osseous points, and at its lowest part was a kind of thick cartilaginous ring, with a depression in the centre containing a cleft. Much loose and putrid membranous matter was washed out by injections of tepid water, the edges of the wound were then brought together by a suture, and suitable dressings applied. After the operation (which occupied three quarters of an hour), two grains of musk in powder were administered every two hours, and a dose of a mixtura acida containing musk was given every hour. In the evening the patient complained of thirst and nausea, and the dressings required to be renewed on account of the profuse discharge. On the following days bark with elix. aromat. acid. was taken alternately with a musk mixture, and port wine diluted and warmed injected daily into the sac. Under this treatment the constitutional and local symptoms progressively improved, the vomiting ceased, the wound secreted healthy pus, and on the 13th of October she was able to leave her bed. The patient who for two years, during which she had borne the child, had continued to menstruate, ceased to do so after the operation, until on the 23d of November bleeding from the wound took place. In the evening there was a discharge of blood from the vagina, and at the same time that from the wound diminished. From this period the catamenia recurred at regular intervals through the natural passages. She is now in good health: the wound in the abdomen is still open, though the discharge from it is inconsiderable.

The writer is of opinion that the fœtus lay in the cavity of the uterus, that the cartilaginous ring at the inferior part of the sac, was nothing else than the cervix uteri, and the depression in it the orificium internum. Several circumstances, he thinks, corroborate this view of the case: from the commencement of the pregnancy, the abdomen was always uniformly distended, behind the sack nothing could be felt but the vertebral column, and after the operation the neck of the uterus ascended, and the os tincæ was firmly closed. The impossibility of birth in the natural way, he attributes to the cartilaginous state of the ring, in consequence of which the inferior part of the uterus was incapable of dilatation.—*Siebold's Journal für Geburtshülfe, Frauenzimmer und Kinderkrankheiten. Band* XV. 1835.

*Proposal for Removing Mother's Marks.*

*By Dr. Pauli, of Landau, in Rhenish Bavaria.*

Dr. Pauli believes from the result of some experiments, that these unseemly discolorations may be removed by *tattooing*, and suggests the following method of performing this operation: The affected place is to be washed with warm water and soap, and then rubbed a little. The skin thus rendered tense, is to be smeared over with a colour (made of white lead and cinnabar), corresponding to that of the surrounding healthy skin, but of a tint somewhat brighter, and it is now to be repeatedly pierced with three needles tied together. They are to be introduced in a rather oblique direction, and so as to produce a little bleeding, and from time to time they should be dipped in the colouring matter. The extent of surface tattooed at a time should at first be small, otherwise there is much inflammatory swelling of the part. The pain is trifling. Dr. Pauli will, at a future time, communicate the result of his experience in this method of removing nævi.—*Siebold's Journal für Geburtshülfe, &c.*

---

STATE OF THE MEDICAL PROFESSION IN FRANCE, SINCE THE FIRST REVOLUTION TO THE PRESENT TIME.

One of the acts of the Revolutionary Destructives of France, in 1792, was the abolishing of all the existing universities, faculties, and learned corporations. Among the rest, the medical colleges were suppressed, and men educated and uneducated, licensed and non-licensed, were all and equally permitted to practise physic and surgery. Every town and village swarmed with the most impudent quacks, " qui distribuaient les poisons et la mort avec une audace que les anciennes lois ne pouvaient plus reprimer."

It was soon found by the government that this state of monstrous confusion could not with safety be permitted to exist, and that the old system, although full of defects, was infinitely preferable to the " mauvaise egalité," which had superseded it.

At the period of the abolishing act, there were no fewer than 18 medical faculties in the kingdom. Of this number indeed, nine only, those of Paris, Montpelier, Toulouse, Besançon, Perpignan, Caen, Rheims, Strasbourg, and Nancy, had for many years been efficient; and of these, the two first had long taken the precedence of all the rest; the remaining half, " n' avaient plus qu'un vain nom." All these eighteen faculties enjoyed the privilege of conferring degrees, and of admitting the graduates to medical honours. Besides them, there were other colleges of medicine in some of the principal towns; viz. at Amiens, Angers, Bourdeaux, Chalons, Clermont, Dijon, Lille, Lyons, Moulins, Nancy, Orleans, Rennes, Rochelle, Tours, and Troyes. These subordinate colleges, not having the right of delivering public instructions, and of conferring hohours, could be viewed only as corporations, empowered to bestow certain municipal privileges on those who practised within their immediate jurisdictions. The course of study prescribed at the different faculties throughout the kingdom was regulated by rules and laws which issued, either from the supreme council of state in Paris, or from the local authorities. The pupils were obliged to study for three or four years; and when candidates for degrees, they were subjected to public examinations, and were required to write and defend theses which had been assigned to them, by the professors.—The expenses attending the graduations did not exceed in all from 400 to 600 francs at the provincial schools, and 6000 francs at Paris. Besides these charges, there were certain fees, amounting to 100 or 150 francs, for their annual " inscriptions," during their residence in the metropolis.

The system of education, and the mode of obtaining honours, now mentioned, may probably be regarded as very satisfactory and highly judicious; and so it would have been, had the spirit of the enactments been truly acted upon; but abuse upon abuse had crept into every part of every institution, and had converted the " fair seeming" into a hideous reality.—There were "domini intra-muros," and "domini extra-muros;" there were "ubiquistes," and "bacheliers," "licencies," "agregés," "docteurs-regens," and "non-regens." In short, so utterly pervaded had the whole system become with the spirit and devices of priestcraft, that the grades of the medical profession resembled rather the orders of a priory, or of a monkhood, than the natural divisions of a society of men, who were to be engaged in constant intercourse with the world. Under the pretext of maintaining the discipline of the "corps," the members were watched, warned, and not unfrequently prosecuted, not only for heresies of medical opinion, but also for their theological tenets, and for their conduct in private life. It is not yet forgotten by some, what wars and rumours of wars were excited by the introduction of antimony as a drug, by the practice of innoculation, by surgeons daring to practise medicine, &c.: and so remiss and corrupt were many of the faculties, that all, with the exception of those at Paris and Montpelier, had become such easy almæ-matres for "recipiendaires" or candidates, that it was no unfrequent occurrence, that a license, granting the "summos honores artis medicæ" was sent off by the post to an aspirant, who had never visited the place.* But these abuses, though worthy of deep reprobation, were not so bad as the chaotic confusion introduced by the Egalité men during the revolutionary madness, and which continued from 1792, until March, 1803, when an entire reform was once more felt to be essentially necessary. Like almost all the other legislative acts of Napoleon, the new laws, which were then introduced, were stamped with the impress of a most comprehensive and wise spirit; for while they still continued the existing privileges to those medical men, and midwives who were in actual practice at the time, all future aspirants were obliged to pass through a rigid course of study, and were subjected to severe examinations, before being admitted to the right of practising any branch of medicine.

Six colleges, at Paris, Montpelier, Strasbourg, Bourdeaux, Lyons, and Rheims, were instituted, and recognised as public government institutions, where full courses of medical lectures were appointed to be annually given; and which were invested with the power of receiving students, and conferring degrees of doctorate in medicine and surgery, "professions qui ne peuvent plus etre separées, depuis que leurs etudes sont fondées sur les memes bases et sur les memes principes." The candidates were to be examined, at distinct and separate ordeals, in anatomy and physiology, in pathology and nosology, in materia medica, chemistry and pharmacy, in hygiene and legal medicine, and on clinical cases, which were to be either "externe ou interne," according as the candidate aspired to a doctorate in surgery, or in medicine. The most objectionable part of the new system was the establishment of the "officiers de santé," a class of sub-educated and sub-privileged medical practitioners, resident in the villages and smaller towns, and who were permitted to attend in the less-important (or rather we should say, in what were wrongfully *deemed* the less-important) cases, and to perform only the minor operations of surgery and midwifery, unless a doctor was present and sanctioned his performance of the more serious operations. The candidates for this grade were, after serving six years "auprés des docteurs," or five years in some military or civil hospital, to be examined by a medical jury, established in each department of the empire, and which was composed of several doctors residing within its limits. Besides the two classes of medical practitioners, viz. the regular doctors, and the officers of health, the law of March 1803, recognized a third class, viz. that of the

* The corruptions of one country will generally find their counterparts in those of another: "eadem facies, eadem origo." Some of our Northern brethren will, no doubt, recognise where the family resemblance lies.—Rev.

midwives; who, before they were authorized to practise, were required to have attended two annual courses of instruction at a public hospital, and to have passed certain examinations held by the same juries, as had the power of admitting the officers de santé. On no occasion, however, were the midwives to employ instruments in cases of difficult labour, unless with the permission of a doctor, who had visited the patient.

Such is a very brief sketch of the law which came into operation about the end of 1803. Hitherto we have not alluded to the remuneration of the professors in the recognized schools. This was to be derived partly from a government grant, and partly from the produce of fees for inscriptions, examinations, and admissions of the students. The former amounted to 3,000 francs to each professor, and was paid from the bureau of the Minister of the Interior. In addition to this public payment, there was an annual grant to each of the colleges, for their incidental expences, such as for the support of their libraries and museums, the wages of servants, and soforth; 40,000 francs were allowed to the school at Paris, 30,000 to that at Montpelier, and 20,000 to that at Strasbourg.

These three schools received, in 1808, the title of Faculties, and were made subject to the Academies, in whose district they were placed. No important change in their organization took place, until November, 1822, when, in consequence of some disturbances in Paris, the Metropolitan Faculty was suppressed by the following Royal Ordinance.

" Louis, &c.

Considering that scandalous disorders occurred at the annual sitting of the Faculty of Medicine of Paris, on the 18th day of the present month, and that it is not the first time that the students of this school have been led into conduct which may be dangerous to the public peace; considering also that the imperative duty of the professors is to maintain a proper discipline, without which instruction cannot produce any benefit, and that the late events indicate ' un vice interieur,' in the organisation of the Faculty, we have ordained, and do ordain, that the Faculty of Medicine be suppressed, and that our Minister of the Interior shall present to us a plan for its re-organisation, &c. &c." In the following year, the remodelling was carried into effect. The faculty was made to consist of twenty-three professors for the various branches of learning: viz.—Anatomy, physiology, medical chemistry, medical physics, medical natural history, pharmacology, hygiene, surgical pathology (two professors), medical pathology (two professors), operations and apparatus, therapeutics and materia medica, legal medicine and midwifery, clinical medicine (four professors), clinical surgery (three professors), and clinical midwifery.* Attached also to the Faculty was to be a certain number of resident " agregés," or associates, whose duty it was to supply the place of the professors, in the event of their being prevented by sickness, or other cause, from fulfilling their duties. The associates were to be selected from those doctors of medicine, or of surgery, who had reached their 26th year, and they were arranged in different grades, according to their talents, and the periods of their service—" la durée du stage est de trois ans; celle de l'exercice de six ans; ceux qui l'ont terminé deviennent agregés libres."—The professors were to be appointed, for the first time at least, by the Crown, and two thirds of the agregés by the High Steward (le grand maitre): the other third was to be nominated at a concours held at the close of the first session. The election of a professor at any future time, was appointed to be made thus: three candidates, who must be agregés, were to be proposed by the Faculty, and three by the Council of the Academy; and the nomination was

* Since the date of this Royal Ordinance, other two Regius Professorships have been instituted; viz. a fourth one of clinical surgery (in 1829), and one of general pathology and therapeutics, (in 1831).

to be vested in the High Steward. The Dean or President of the Faculty, was to be appointed by the same functionary, (the office of dean to be held for five years); and also the inferior officers, viz. the librarian, conservator of the museum, chemical assistants, clinical chiefs, chef des travaux anatomiques, prosecteurs, and aides d'anatomie.

In order that the regular attendance of the students might be assured, they were required to inscribe their names in a register (prendre inscription), during the first fortnight of each "trimestre;" but it is to be observed, that to be entitled to a "carte d'inscription," they were called on to produce certificates of their births, of their good conduct and manners (from the mayor and prefect of the "commune,") and also diplomas of bachelors of letters, and of sciences.*

At the end of every trimestre, it was ordered that a report of the proceedings of the Faculty should be made by the Dean to the Rector, and by the latter to the Lord High Steward, for the information of the Government. All reprehensible conduct, whether on the part of the professors, agregés or pupils, was required to be forthwith reported to the Dean, whose duty it was to take cognizance of it, and to inforce the immediate corrective. In reference to the former two classes, the 30th art. declared, "Tout professeur, tout agrégé, qui dans ses discours, dans ses leçons, ou dans ses actes, s'ecarterait du respect dû à la religion, aux mœurs, ou au gouvernement, ou qui compromettrait son caractere, ou l'honneur de la Faculté par une conduite notoirement scandaleuse, sera deféré, par le Doyen, au Conseil Academique, qui, selon la nature des faits, provoquera sa suspension, ou sa destitution, conformement aux statuts de l'université."

It will be perceived from the preceding details, that the entire regime of the French Faculty of Medicine was thus essentially subjected to, and ruled by the executive government of the state. We see that, by the fiat of one Royal Ordinance, the whole existing system may be at once abolished, and created anew at the will of the king and his ministers. When the new code was announced, there appeared at the same time a Royal Decree, nominating professors to the different chairs of instruction. Eleven of the former professors were re-appointed, and nine others were selected in the room of those, who by their conduct had drawn down the censure of government.

Such was the state of things from 1823, to the expulsion of Charles X. from France.

But, after the revolution of July 1830, Louis Philippe undid what Louis XVIII. had ordained; for he restored the expelled professors to their chairs, and excluded those who had been appointed by the ordinance of 1823 in their place.

All future appointments were ordered to be made by concours. The faculty of medicine, which had been abolished in 1822, was re-instituted with all its former privileges and honours.

The administration of the medical, and indeed of all other seminaries of education throughout France, is vested in the "ministre de l'instruction publique et des cultes."—*Jurisprudence de la Medicine, &c. en France.*

---

### Notice of some of the English, German and Italian Medical Museums. Cursory Remarks on Italian Medical Literature. Acknowledgment of Books received from Dr. Warburton of Palermo, &c.

Dr. Martins, during his visit to England and Germany in the course of last year, drew out a short description of some of the best anatomical museums in both countries, and communicated it to the pages of the Revue Medicale. He men-

---

* By a Royal Ordinance, dated 18th January, 1831, the scientific graduation was to be no longer a requisite preliminary.

tions with praise the small, but "admirably arranged" museum at Oxford, and particularizes, as especially worthy of notice, a most beautifully injected preparation of the human testicle, presented by Sir A. Cooper,—and the Lilliputian-made foot of a Chinese woman: its extreme length, from the os calcis to the point of the great toe is four inches, and from the os calcis to the point of the little toe, only two inches.

In the museum, attached to St. Thomas's Hospital, one of the most interesting preparations which he saw, was a skeleton, exhibiting a remarkable universality of tuberculous deposition: the trochanters, the vertebræ, the sternum are affected with it; and of the soft parts belonging to the subject, the lungs, uterus, liver, pancreas and mesenteric glands, mammæ, integuments of the chest, the pectoral and intercostal muscles, the pleura and pericardium, and many of the lymphatics, exhibit traces of the same morbid change. In the same museum are preserved numerous valuable preparations, illustrating the phenomena of aneurism. There is one of an aneurismatic aorta opening into the jejunum; and another of a femoral artery, where the aneurismatic tumor had compressed the vessel immediately below, or plantad to itself, and had thus brought about a spontaneous cure of the disease. Among the specimens of diseased veins, there is one of the internal jugular, which has become so much distended, that the varicose swelling is nearly as large as a hen's egg.

At Guy's Hospital Museum, Dr. Martins' attention was directed more especially to the beautiful and valuable collection of drawings and wax models illustrative of cutaneous diseases. One of the wax models represents with admirable fidelity the rare disease, pompholix; the bullæ vary in size, from that of a pea, to that of a pigeon's egg; they are represented as filled with a reddish serosity. The disease had supervened on an attack of paraplegia, the result of fractured vertebræ. Dr. Addison, one of the physicians of the hospital, and who has been a pupil of the late Dr. Bateman, is entitled to great praise for his valuable additions to this department of the museum.

We regret that Dr. Martins has taken so short a notice of that museum, in some respects the most extraordinary one in the world, which was made by the late John Hunter, and now constitutes the museum at the College of Surgeons in London.

The labours of British anatomists, in the field of comparative anatomy, are but very little known on the Continent; and although we must confess that of late years they have been, when compared with the deeds of Cuvier and of others in France and Germany, but as the Arab hut beside the mighty pyramid, it is ever a proud satisfaction in our humility to lean back upon the bulwark of Hunter's name. The almost incredible achievements of this one man have been, hitherto, almost a sealed book, not only to foreigners, but to most of his countrymen; and we cannot therefore wonder much that in the historical reviews, or sketches which have of late years been so frequently and ably drawn up by many French and German authors, the value of the Hunterian Museum has been so imperfectly appreciated.

It is to be hoped that the labours and discoveries of Hunter may be henceforth better known and prized, now that not only a copious and descriptive catalogue is publishing by the College of Surgeons, under the able superintendence of that excellent anatomist Mr. Richard Owen, but also a new edition of Hunter's works (which is to contain in addition to all his published writings largely annotated, numerous manuscripts and detached memoirs, as well as the substance of Sir E. Home's Lectures on Comparative Anatomy, which, it is well known, were drawn from Hunter's notes) has been promised to the medical world. To recur to Dr. Martins' notes, we find him stating that to examine the Hunterian Museum in detail, "il faudroit lui consacrer au moins trois semaines." This accidental remark is more highly laudatory, than the most laboured eulogy. He notices in particular the skeleton of the gigantic Irishman who was eight feet in height; the preparation of the pelvis of the negro woman, on whom Dr. Steevens performed successfully the operation of tying the internal iliac; the thorax of a patient who recovered after the frightful accident of a carriage-shaft having been driven fairly through it, from one side to the other; and the preparations of the genital and mammary organs of those singular animals, (whose proper place in the zoological scale has been such a perplexing problem) the ornithorynchus paradoxus, and the echidna hystrix, which have been at length satisfactorily proved by Mr. Owen to be truly mammiferous.

The German museums, which Dr. Martins visited, were those of Heidelberg and Strasbourg. The former has of late years been splendidly enriched by the labours of Tiedemann, Arnold, Fohmann, &c. The preparations of the nervous system are extraordinarily beautiful; and of these, more especially some which display the "ganglion oticum, seu Arnoldi," and others which shew the nervous supplies of the uterus.

In several preparations of the lymphatic system, the mercury has passed from the mesenteric lacteals into the branches of the vena portæ: this phenomenon has been noticed in the human subject, also in the seal, in several dogs, and in the large tortoise. We are told "that all the intestinal lymphatics seemed to be collected at the spleen, which was thereby quite covered with a mercurial network." These preparations tend to confirm the opinion of Tiedemann, that the spleen is to be considered as a huge lymphatic ganglion, whose office is to assimilate the chyle with the blood.

At the Strasbourg Museum there is a curious preparation, which exhibits the presence of four spleens in one subject. M. Lauth mentioned to Dr. Martins that in all his experiments of removing the spleen from animals, there was a new supplementary one formed, or forming, provided the animal survived, for a sufficient length of time, this severe operation. If this statement be correct, the hypothesis of Tiedemann certainly seems to be not improbable.

The anatomical museum of Strasbourg owes much to the exertions of Professors Lobstein, Lauth, Ehrmann, and Aronsohn: the pathological department is especially rich and valuable, and is now one of the most complete collections in Europe. There is a remarkable specimen of a sternum bifurcated in its upper half; the clavicular articulating surfaces are at least three inches and a half apart from each other. Among the vascular preparations, there is one of the arch of the aorta being bifurcated and again re-uniting; the trachea and œsophagus pass between the limbs of the bifurcation. Dr. M. says, "il n'existe que deux exemples de cette disposition; celui du musée de Strasbourg se rapporte à l'année 1777." Among the intestinal preparations, one of a very extensive invagination is especially worthy of notice; the extremity of the ileum, the cæcum and its valve, and also the transverse part of the descending colon are introsuscepted within the rectum, so that the vermiform appendix of the cæcum is not more distant than two, or three inches from the anus, and had been supposed, during the life of the patient, to have been a polypus recti. In another bottle, there is preserved a portion of the small intestines, fully a yard in length, which had been passed by the rectum after an attack of ileus: the gut is of a dark colour, but is not gangrenous. The patient, a young girl, recovered completely from the attack; but unfortunately four months after the cure, she eat a quantity of cherries and their kernels, and a violent colic, with most severe tormina, was induced: the intestinal cicatrix gave way, and, the contents of the bowels escaping into the abdomen, death speedily followed.

To the preceding short notes drawn from Dr. Martins' paper, it may be interesting to subjoin a few of the observations on the Italian medical schools, made by M. Roux in his late visit to the South. The anatomical museum at Pavia, he says, is rich and extensive, and is especially valuable for the numerous beautiful preparations, by the late Professor Scarpa, of the minute anatomy of bones, of the parts concerned in herniæ, and of the arterial anastomoses in healthy and in diseased conditions.

The admirable preparations of the nerves and lymphatics made by M. Panizza* (an anatomist worthy of being the successor of his great countryman Mascagni) must arrest the attention of every stranger. One of these exhibits a beautiful demonstration of the mode of connexion between the filaments of the great sympathetic nerve, and the spinal nerves. What however attracted M. Roux's notice in an especial degree, were several preparations of the blood-vessels of serous membranes.

It is generally known that many anatomists have questioned the presence of blood-vessels in these membranes, and that they have considered them as a sort of epidermis, under which the vessels and nerves are situated. "I confess," says M. Roux, "that I was always unwilling to assent to this doctrine, although I could not satisfactorily to my own mind disprove its correctness. Since I have examined preparations in the museum of Pavia, all my doubts have been completely cleared away. Some of these preparations exhibit the serous membranes unravelled in the most curious manner, and penetrated with blood-vessels filled with fine injection. The vascularity of these membranes is henceforth, to me at least, a fact of incontestable demonstration."

With respect to the collections of pathological anatomy in Italy, M. Roux talks of many in terms of very high approbation. He takes occasion, at the same time, to reprove his own countrymen for their remissness in not preserving the numerous specimens of morbid changes, which are daily examined in the large hospitals of Paris. It is a fact perhaps not generally known, that there is not in France a pathological museum equal in richness and extent to several private ones in England, Germany and Italy. We believe that the one at Strasbourg is the most extensive and the best arranged of any in France.

M. Roux is entitled to much praise for his candour, in avowing a national failing, and in admitting the superiority of strangers; and we have the more pleasure in alluding to his conduct in this respect, because it is of so very rare occurrence among his countrymen. His words are; "in Italy pathological anatomy is cultivated with much less egotism, than with us in France. We have indeed done much for science, but very little for those who pursue it, and who may wish to verify recorded facts. Nowhere have we any collection that deserves to be mentioned at the same time with many in foreign countries; and it will take a considerable time, and not a little labour, before we can hope to vie with most of our neighbours in this respect."* The museum at Florence contains some very curious preparations. There is a cranium, which had been so fairly transfixed with a dagger, that its point projected nearly an inch beyond the inner table of the bone. The patient survived for a long time, "portant le fer enclavé dans le crane."

There is also the uterus of a woman, who had undergone the Cæsarian operation twice. She had died in consequence of the second. M. Roux makes some remarks on the state of surgery in Italy, and expresses an opinion that there appears to be a reluctance to adopt many of the improvements, which have of late years been

* We propose in our next number to call the attention of our readers to the discoveries of this excellent Italian anatomist, in our review of M. Breschet's recent work on the Lymphatic System.—*Rev.*

* Since this was written by M. Roux, the museum founded and endowed by the late Baron Dupuytren, has been established in Paris. It already contains many valuable pathological preparations, and it may speedily be made worthy of the French school of anatomy, which has deservedly stood so high for many years. It would be difficult to match such names as Bichat, Cuvier, Beclard, Cloquet, Cruveilhier, Andral, &c. in any other country.

introduced into this branch of the healing art by the French school.—Thus lithotrity has been practised only once, by a surgeon at Florence. The doctrines and precepts of Scarpa are almost exclusively paramount in every school; and seldom is a deviation made from the treatment which this distinguished surgeon has inculcated in any particular disease. Thus the operation of extracting the cataract is seldom or never attempted under any circumstances; Scarpa's mode of couching being almost invariably adopted.

We must here take a rather abrupt leave of M. Roux and his rambling notes; but before quitting the subject of Italian schools, we are anxious to fulfil a duty, which it has been long our purpose to do—viz. to acknowledge with many thanks the receipt of several works, published at Palermo in Sicily, from Dr. Warburton resident there.

Without further apology, we introduce the 'ipsissima verba' of the short notice of them, which we drew up many months ago.

We received lately, from Dr. Warburton of Palermo, the following works published there within the last two or three years; the first vol. of a Corso Completo di Anatomia Descrittiva, by Signor Gorgone, Professor of Anatomy in the University; a pamphlet on the preservation of subjects for dissection; two clinical surgical reports; a lecture on fungus hæmatodes, or encephaloid tumor,—all from the pen of the same gentleman.

We are also indebted to Dr. W. for a copy of the Regulations of the Royal Lunatic Establishment, founded a few years ago at Palermo, and two memoirs on subjects connected with insanity, by Dr. Silvestri.

Our best thanks are due to Dr. Warburton for his kind attention in sending for our inspection the above works, and we have only to regret that they did not reach us sooner.

There is a great difficulty in procuring Italian medical books in London, or we should take more frequent opportunities of communicating to our readers the state of professional literature in a country which may boast of such distinguished men as Morgagni, Baglivi, and Scarpa. It is, therefore, especially gratifying to us to receive from our readers abroad any of the productions of the foreign press. Some of our cotemporaries have of late been making a prodigious parade of their deep read lore in all the living languages of Europe, and not satisfied with the French, German, and Italian journals, they have been ransacking (at least they tell us so), the Russian, Swedish, Danish, &c. periodicals to serve up some marvellous tale upon subjects never heard of befoie in this country.

We do not profess to be such universal linguists, and perhaps our readers would not thank us for regaling them with mere frothy announcements of the titles of books, without telling them any thing of their contents. Our aims and efforts are very different.

The communication of all important physiological and pathological discoveries, and of all substantial improvements in practical medicine, is what we aim at doing—this is the great object of our labours. As a matter of course, our own literature will always receive the chief attention from us, not only because it is quite natural that we should be more interested in domestic than in foreign productions, but also because we are well aware that English thoughts and English sentiments will always make a greater impression on English readers, than the thoughts and sentiments of foreigners. The English are so truly and so strictly utilitarians in all they do, and in all they think of, that unless something tangible, something which can be proved to be true, and which may lead to practically beneficial results, be presented to them, they quickly turn away from the displays of book knowledge, however ingenious and recondite. It is the solid substantial food which they ever have preferred, and always will prefer, to the elegant trifles and souffléed confitures of continental production. In justice to the Italians we must confess, that the little which we have read of their medical productions has, in some respects, left a more favourable impression on our mind, than that which we have of either the French or the German literature. There seems to be less of wearisome detail about unimportant matters, and less proneness to vague theorising in their writings.—The almost unequalled excellence of the Italians in some branches of science, of art, and of literature, might lead us to anticipate the most pleasing hopes of their success in any department of philosophy to which they will direct their attention; and we verily believe that it requires only the vigour and energies of the Italian mind to be liberated from the stupifying enthralment of a bigotted superstition, and of an illiberal despotism, for it to assume, perhaps, the foremost rank in the investigation of medical as well as of other truths.

Gifted with more lively perceptive, and more richly imaginative powers than the English, but inferior probably to them in patient industry and in calm reflection, the Italians are free from the transcendental mysticism of the Germans on the one hand, and from the ever-changing credulity of the French on the other. We should gladly embrace every opportunity of studying the writings of the Italian physicians, and of communicating the results of these studies to the British public, if there was a greater facility of procuring them; but they are really not to be had in our London shops, and when ordered from Paris, we have been sometimes obliged to wait for upwards of a year before we have received any answer. We need not, therefore, repeat our assurance to Dr. Warburton, and to others similarly situated, that we shall ever regard it as a great favour, if they will occasionally transmit to our care any of the more esteemed works published in the countries, where they reside.

The first volume of the Corso Completo di Anatomia, is occupied with an account of the bones and ligaments. The anatomical details are minutely and very faithfully given, and great attention has been paid to the description of the various changes which each bone undergoes from early infancy to mature age. As this is quite an elementary work, it cannot be expected that we should give an analytic review of its contents.

If our opinion is worth aught to Dr. Gorgone, we may assure him that we have read the first volume of his anatomy with much pleasure, and that we regard it as an excellent manual, full of correct and valuable descriptions. We have introduced some of the practical contents of the shorter works in our subsequent pages, under that division of pathology to which they may appertain.

---

### On the Chemical Properties of the Secretions in Health and in Disease; and on the Existence of Electrical Currents in Organized Bodies, induced by the Acidity and Alkalinity of their different Membranous Surfaces.

M. Donné, whom we have had repeatedly occasion to mention with praise, is the author of some curious—would that we could add, well-ascertained—statements on this subject.

All that we propose to do, is merely to present to our readers the leading results of his enquiries. They are contained in the following corollaries.

1. The whole of the tegumentary surface secretes an acid humour. It is however to be noticed that the sweat, instead of being, as it is generally stated, more acid in the axilla, and around the organs of generation, than in other parts, is frequently of an alcaline character.

2. The alimentary canal, from the mouth to the anus, except the stomach, (the gastric juice of which is strongly acid, as has been proved by Prout, Tiedeman, and Gmelin,) secretes an alcaline mucus. Thus the saliva, and also the mucus of the pharynx and œsophagus as far as the cardia, and of the intestinal canal from the pylorus to the anus, are alcaline in health; and become acid, only in consequence of disease.

3. The serous and synovial membranes secrete an alcaline fluid: in disease, it sometimes becomes acid.

4. The external acid, and internal alcaline membranes of the body, represent the two poles of a galvanic pile, whose effects are appreciable by a galvanometer. For, if one of the conductors of this instrument be placed in contact with the mucous membrane of the mouth, and the other conductor be applied to the skin, the magnetic needle will be found to shew a deviation of from 15 to 20, or even 30 degrees; and the direction of the needle proves that the mucous or alcaline membrane indicates a negative electricity, and the cutaneous or acid membrane a positive electricity.

5. Independently of these two great surfaces, exhibiting opposite electrical states, there are other smaller cognate systems, which are similarly opposed. Between the stomach, for example, and the liver, we may discover energetic electrical currents.

6. The acid humours of the system may become alcaline, and the alcaline may become acid, in a state of disease.

7. An abnormal acidity is usually the result of a phlegmasia; and this change may take place in an organ, at a distance from the inflamed part;—thus the saliva becomes strongly acid in gastritis.

8. The acid, developed during the existence of inflammatory disease appears to be most frequently the hydrochloric. The presence of this acid may very possibly determine the coagulation of the albuminous part of the lymph, or serosity, which abounds in all inflamed structures; and we know that this coagulation is the cause of the false membranes in the inflammation of serous membranes, of specks and opacities of the cornea, and of the induration and hypertrophy of many parenchymatous organs.

Purulent matter is produced by the action of an acid upon albuminous lymph:—it is a species of combination of acid and of albumen. Although we cannot always discover traces of a free acid in inflammatory effusions, and although pus does not always redden the blue paper of turnsol, we are to remember that by far the greater number of the humours of an animal body in health are strongly alcaline, and that in this way the generation of acid in disease may be masked or concealed for some time, in consequence of the neutralising effects of the original or primary alcali.

9. The alterations in the chemical nature of the secretions must necessarily react on the different functions of the system. They will be found to constitute an interesting groupe of lesions, or symptoms, hitherto but little regarded, and the diligent investigation of which may very possibly lead to some important therapeutic results.

These changes will probably be found to induce certain modifications in the electrical currents, which exist between the different organs of the economy.

---

### Researches on some of the Properties of Pus and of Blood—Mode of distinguishing the Globules of these two Fluids—Effects of Admixture of Pus with the Blood.

M. Donné has contributed a very ingenious and valuable memoir on the above subjects to one of the most esteemed of the French Journals. It merits an attentive perusal from the pathologist, as some of its statements tend to elucidate the history of those very obscure cases, in which the mass of blood appears to be, as it were, poisoned by the admixture of purulent matter, and in which there is a marked tendency to the deposition of pus in some inward parts, such as the parenchymatous viscera, the joints, &c. without any previous inflammation of these parts. To this state of the blood, M. Piorry has recently given the name of "pyohemie," or "entorrhée pyohemique." We shall make a few extracts to exhibit the spirit of M. Donné's memoir.

Pus, whatever be its colour, consistence, and smell, has always exhibited in my experiments the following character:—

1. Treated with concentrated ammonia, it is converted into a transparent and very tenacious jelly. A small portion of pus, when mixed with a considerable quantity of water, continues to shew this property. Sometimes it takes a short time before the change is complete; but by stirring the fluid with a glass rod, the gelatinous transformation is speedily apparent. I have never failed in obtaining this result with all the varieties of pus. A watch-glass is the most convenient vessel in which we may perform the experiment.

2. Examined with the microscope, pus, whatever be its character, appears as a fluid in which there floats a number of globules, all of the same size, and all having a diameter nearly double of that of the blood-globules.

When the pus becomes decomposed and putrefied, the globules separate from each other, and at length they appear to be dissolved; but this change takes place very slowly and tediously—six or eight days at least are necessary. That the globules of purulent matter are not dissolved by the action of the strong ammonia, may be readily ascertained by the microscope.

The property of pus, to be converted into a gelatinous substance when treated with concentrated ammonia, has been noticed by M. M. Andral, Gendrin, and others; but these authors do not seem to have attached to this property, as a diagnostic sign or indication, the importance which it deserves. We are inclined to believe that, by this character alone, we may almost always distinguish pus from every other animal fluid. Thus the serum of the blood, the saliva, the bile, the urine, a solution of albumen, &c. do not exhibit it; whereas a very small quantity of pus mixed with water becomes, on adding some drops of ammonia, either immediately, or after the lapse of a few minutes, ropy and of a glairy consistence, not unlike to the white of an egg, or to one kind of bronchial sputa.

Although in the present day we do not attach the same importance to the discrimination of pus from mucus, which physicians were some years ago in the habit of doing (when it was supposed that the presence of pus is always indicative of actual ulceration), yet it must be very desirable to be possessed of a means of distinguishing the one fluid from the other. M. Donné assures us that he has always succeeded in his diagnostic trials by using the above test. He introduces some of the sputa to be examined into a glass tube, closed at one end, adds a small quantity of water, and shakes the mixture well together.

It is worthy of notice, that this simple experiment alone will often suffice; for, as M. Gendrin observes, "of all the chemical analyses of pus, that which is most important and which best deserves our confidence, consists in the mere dilution and agitation with pure water—the pus gradually subsides, and the yellowish pulverulent (?) substance (la substance pulverulente jaunatre) which is precipitated, has all the physical properties of pus. But the value of this experiment is greatly enhanced, if we conduct it in the following manner. Put some of the diluted pus into a watch-glass, and add a few drops of concentrated ammonia: the appearance of the gelatinous change is immediately, or very speedily obvious. If the sputa contain no pus, but mucus only, the fluid remains ropy—the ammonia having no power to destroy the viscidity of mucus—but it does not become glairy and tenacious, as it does when pus is present.

M. Donné says that in some cases of diseased urinary organs, the admixture of semen with the urine may simulate the appearance of purulent matter. The ammonia renders the semen less viscid than it is in its natural state; but the best test for discriminating the one fluid from the other is the use of the microscope. The form of the spermatic animalculæ is quite peculiar and characteristic. A case in point occurred in the clinical wards of La Charité lately. A quantity of milky urine was taken from the bladder of a man who had died from encephaloid cancer of the stomach and bladder, and who had experienced considerable dysuria. It was doubtful whether this whitish urine contained pus or not. Examined by the microscope, a very large quantity of dead spermatic animalculæ was at once discovered, and when strong ammonia was added, no gelatinous change took place.*

With respect to milk, when treated with strong ammonia, it is worthy of notice, that its globules are not at all affected by it; and this negative property affords a useful and easy test to separate and to distinguish milk from blood or serum, when it happens to be mixed with either of these fluids—the blood globules being at once dissolved by the strong ammonia, and the milk globules remaining intact. The milky fluid, which is secreted during the first day or two after delivery, and which is known by the name of "colostrum," has a greater analogy, in its relations with ammonia, to pus, than any other animal fluid which M. Donné has examined. When the volatile alkali is added to it, it immediately becomes viscid and tenacious, and is converted into a glairy mass.

The serum of the blood, separated from the coagulum, is little, if at all affected, at least visibly, by the ammonia. But the blood itself, when it has not yet separated into its component parts, and is still a homogeneous fluid, is acted on by ammonia in a manner very similar to that which the alkali exerts on pus: at least this test does not enable us to distinguish blood containing pus, from blood which is pure and free from admixture. Blood, when treated with strong ammonia, is changed into a viscid jelly, not unlike to red currant jelly, The circumstance, therefore, of blood and pus being similarly affected by ammonia, renders the separation of these two fluids a very difficult problem. The microscope fortunately here lends its assistance. The size and form of the globules of pus is very different from the size and form of the blood-globules—the former are larger, have no central nuclei, are not flattened, but are altogether globular or spherical, and their edges are less even and smooth, than we find to be the case with the globules of the blood. To add assurance to this experiment, it will be always prudent to try the ammonia test on the fluids under the microscope, as recommended by Müller in his Memoir on the Blood, published in the Annales de Physique et de Chimie de Berlin, 1832. The strong ammonia (or a solution of caustic potass) attacks not only the colouring envelopes of the blood-globules, but also dissolves their nuclei. We have already said, that the pus globules are not affected in this manner.

In examining the state of the blood, M. Donné recommends the physician to conduct his experiments in the following manner:—Put a drop of the blood, to be analysed, between two thin plates of glass, and examine it attentively with a microscope. If the globules are uniform and alike in every respect, we may conclude that the blood is free from any foreign admixture. On the contrary, should we observe any globules of a larger size and of a different shape from the others, our attention should be directed to ascertain their true nature. The irregular globules may be either globules of pus, or merely some of the blood-globules, which have become changed in consequence of commencing putrefaction, or of some other cause, which at present we do not well understand. In these circumstances, the application of some strong ammonia will solve the difficulty. If there be no admixture of any pus globules with those of the blood, the ammonia causes all the globules to disappear instantaneously, and nothing now is seen between the glasses, but a few straggling filaments, probably of fibrine. If, on the other hand, there are any pus-globules, these remain quite unaffected by the ammonia.

M. Donné closes his memoir by alluding to the effects which the admixture of pus with fresh blood has in inducing a dissolved and diffluent state of the coagulum. The blood coagulates

* M. Donné very justly remarks that the examination of animal fluids, morbid and healthy, with the microscope, has been hitherto too much neglected, and that, if properly conducted, some very useful results might be obtained.

at first nearly as usual; but in the course of a short time—eight, twelve, or twenty hours—the coagulum becomes soft, until at length it is again entirely fluid. The larger the quantity of pus mixed with the blood is, the more speedily do these changes take place.

This solvent effect of purulent matter on the state of the blood deserves to be studied with attention, as it may afford an explanation of some obscure pathological phenomena. We know that, in fatal cases of purulent absorption, the blood is often found to be in a fluid and very diffluent state. So it is also in some malignant fevers, and among these more especially in small-pox. Lister long ago remarked, "in multis autem variolis, quibus urina cruenta mota est, sanguinem e brachio missum adeo aquatum, putridumque esse vidi, ut fluctuaret crassamentum in vase non aliter quam ipsum serum." Perhaps, too, the very rapid formation and deposition of large quantities of pus in certain cases, may be connected with the peculiar property of this fluid to alter and infect the state of the circulating mass, rendering it more liquid, and destroying, in some degree, its tendency to coagulation.—*Archives Générales, Aôut*, 1836.

---

### Extract from a Lecture by M. Serres on some Points in Organology—the Predominance of the Right over the Left Side of the Body.

"The law of the predominance of the right over the left half of the body, is applicable to and is exemplified by all the organisms. Its influence is visible even in the lowest tribes of animals—in the infusoria as well as in the mollusca, and among these, most conspicuously in the gasteropedous family. Pathology also furnishes numerous examples of the operation of this law. For example, supernumerary parts are more frequently observed in the right than in the left side; and, on the other hand, defective formations are more common in the latter than in the former. Among the viscera, the liver exhibits a very marked instance of this inequality of development.

In the early stage of its growth, it is nearly symmetrical, the two lateral lobes not differing much in size; but in the fifth or sixth month of intra-uterine existence, the right lobe begins to acquire that decided superiority of development, which it ever after retains, at least during health. In certain diseased states, this predominance is sometimes lost, in consequence of the two lobes being unequally affected. Thus, in hypertrophy of the liver, we generally find that the enlargement affects the left more than the right lobe. By the result of this morbid action, the primitive form of the embryotic liver is reproduced, and the organ returns to the point whence it started (l'organe revient au point d'ou il etait parti); but it returns under the influence of an operation, which is normal in uterine, but abnormal in extra-uterine existence. In the former case, the state of the liver is one of the conditions of embryotic life; whereas, in the latter, it becomes the cause of death, *because extra-uterine life cannot accommodate itself to the conditions of intra-uterine life*.

It sometimes happens that the organic conditions which appertain to, and which subserve the support of extra-uterine life may be prematurely developed in the fœtus. The consequence of this anomaly is, that the fœtus perishes by the very organic arrangement which is necessary to the continuance of its extra-uterine existence. For example, we may mention the closing of the foramen ovale, or of the ductus arteriosus before birth:—the child dies in the uterus, in consequence of a premature development of certain organisms. In cases such as we have now alluded to, it might, without a minute examination, be supposed that the crime of infanticide had been committed. This subject deserves, therefore, the strict attention of the medical jurist, who, in all that regards infanticide, has need of the steady light of anatomical and physiological science.

But to return to the topic of the predominance of the right over the left side, the following pathological illustrations of this law of organisation may be deemed interesting. Out of 20 cases of coxalgia, 18 will be found to occur on the right side. On the other hand, almost all the examples of spontaneous luxation of the hip have been found on the left side. The same law holds good as regards the tardy descent of the testis. This malformation occurs twenty times at least in the left groin for once that it does in the right. Hitherto surgeons have not attended to such details, as the relative frequency of disease on the two sides of the body; but it is a topic full of interest, and perhaps of practical utility. Fistula lacrymalis is greatly more frequent on the left than on the right side; and the only probable conjecture which I can form as to the cause of this difference is, that there is "une inegalité du developpement des deux canaux nasaux." If we compare a multitude of skulls, we shall find that the left nasal canal is rather more contracted than the right one. This fact affords a rational explanation not only of the greater frequency of the disease on the left side, but also of its more easy curability on the right than on the left side."—*Lançette Française.*

---

### Examples of Double Uterus.*

Before alluding to any of the recorded examples of this structural anomaly, it is proper to premise that two malformations, very different from each other, have been designated under the title of double uterus. There may be either two separate and distinct uteri terminating by different orifices in a common vagina; or only a single uterus divided into two compartments by a longitudinal septum or partition.

Bauhinus has narrated two cases, in each of which the womb contained a double cavity. He, naturally enough in those days of imperfect anatomical knowledge (1599), imagined that the plurality of infants in certain pregnancies may be connected with this irregular formation of the uterus. Riolanus in his Anthropographia mentions that he examined the body of a girl, in whom "ab orificio externo duplici, usque ad fundum uteri, duplex erat matrix, mediano pariete secreta."

One of the most authentic and most accurately narrated examples is that, which occurred in 1681 to Dionis. A young lady died during the sixth month of her first pregnancy. On examining the pelvic viscera, Dionis found that there were two uteri, distinct from each other, but having one common cervix. Conception had taken place in the left uterus, which was found ruptured at its fundus. We are told that this singular malformation was exhibited and explained by Dionis to the Queen and Dauphiness of France, the patient having been one of the women of the royal bedchamber.

It perhaps deserves to be noticed that the catamenia continued in this case to appear during the first four or five months of pregnancy.

M. Littre has related in the memoirs of the Royal Academy of Sciences for 1705 the dissection of a girl, in which the uterus was divided into two distinct cavities, by a septum which extended a short way into the vagina, so that there was a separate opening from the canal into each uterine cavity.

In another case reported in the 5th vol. of Haller's Thesis, the septum did not extend into the vagina: the cervix uteri however was divided into two passages.

In a rare and very interesting work published in 1752 by M. Boehmer, professor of anatomy at Magdeburg, there

* The memoir, from which we draw our account, was written by the late M. Louis, and read before the Royal Academy of Surgery in April 1790, but it has never been published. M. Louis was perpetual secretary of the Academy. Many examples of double uterus have, it is well known, been noticed since the date of this memoir. There is a good description of such a case in the Medical Gazette two or three years ago, by Dr. Lee of London.

is a well drawn up report of a "uterus humanus bifidus et bicornis cum vaginâ duplici." The woman died at the age of 52. She had been married when young, but the consummation of the marriage, we are told, could never be effected, in consequence of the irregular formation of the vagina.

In 1752 M. Bagard published an interesting case of double uterus which he discovered in a woman, who had miscarried fourteen times. On one occasion, she miscarried of twins in the fifth month of pregnancy. There were two distinct uteri conjoined by a common cervix, and opening into the vagina by a common orifice. The two uteri were nearly of the same size.

In Haller's Opuscula Pathologica there is a short account of the dissection of a girl, in whom were found two uteri, each of which was provided with a corresponding vagina. The great physiologist,* in reference to this case, remarks—"Dubium vero non est, quin potuerit in ejusmodi puellâ fieri conceptus post conceptum, atque adeo superfœtatio perfecta locum habere."

In 1772 M. Martin read before the Academy the history of a dissection of a woman 28 years old, in whom he discovered a double opening in the hymen, and two vaginæ, which were parallel to each other, and which terminated in two distinct cavities of one uterus.

In 1781, M. Berthollet mentioned to the Academy the case of a woman, who had borne a mature child 10 years before her death, and in whom he found two distinct uteri; one situated on the right side and the other on the left, but having a common cervix and therefore only one os tincæ. The right uterus was rather larger, and of a more spongy texture than the left uterus, and it was therefore conjectured that the conception had taken place in the former.—*Archives Générales.*

* This most appropriate designation of the distinguished German belongs exclusively now, we are told by M. Bouillaud with a Frenchman's accustomed vanity, to his countryman Magendie! In one of his recent lectures M. B. calls him "le physiologiste par excellence, et le seul qui merite le nom aujourd'hui." Have Tiedemann, Sir C. Bell, &c. shuffled off their mortal coil? —*Rev.*

---

### Example of Triple Kidney in the Human Subject.

A sailor, 39 years of age, died of an adynamic fever in the naval hospital at Oranienbourn. On dissection, the left kidney was found of an extraordinary size, but in respect of situation, colour, consistence, and the insertion of its blood-vessels and nerves, it was perfectly normal. The right kidney, the structure of which was altogether healthy, was situated opposite to its fellow the left one, and rested on the lumbar portion of the diaphragm and the quadratus lumbaris muscle. Its ureter, which was rather smaller than that of the left kidney, passed down as usual to the place where the aorta divides into the two common iliacs. At this point, there was found a third renal gland, which rested on the right iliac artery, and on the psoas muscle. This third kidney was larger than the right one, and was of an oval shape, its two ends being somewhat widened or dilated. Its anterior surface was convex, the posterior one was flattened. The ureter of the right kidney, in passing along a longitudinal groove of this supernumerary one, joined, at about the middle of the gland, its ureter, and the common canal thus formed, and which was wider than either of the regular ureters, passed down to the bladder, and entered it at the usual place on the right side. The third or supernumerary kidney received three arteries; one of these was derived directly from the aorta, another from the right iliac, and the third from the hypogastric artery. One of its veins terminated in the ascending vena cava, and the two other in the right iliac vein. Its nerves were derived from the inferior mesenteric plexus, and they were interlaced with the right spermatic and renal plexuses. The structure of this kidney was altogether similar to that of the other two, in colour and in consistence. The relative size and weight of the three kidneys may be stated thus. The left kidney weighed upwards of eight ounces—the right one between three and four ounces—the supernumerary one upwards of five ounces. The left kidney was four inches and a half long, and nearly three inches wide—the right one measured three inches and a half in length, and nearly two inches in breadth—and the supernumerary one measured three inches and a half in length, and rather more than two and a half in breadth. The right and left kidneys were provided with supra-renal glands; the supernumerary one was not. The bladder and other parts of the uro-poietic system were quite normal. The man had never suffered from any urinary affection.—*Journal fur Chirurgie, &c. von Graefe und Walther.*

---

Discussion at the Royal Academy on Apoplexy and Phrenology —MM. Rochoux, Bouillaud, Broussais, Adelon, Amussat, Capuron, &c.

M. Rochoux is, it is well known by those who are acquainted with the French school of medicine, strongly opposed to many of the recently *devised* (shall we say *discovered?*) doctrines of the physiological school of medicine. He has written much and ably on cerebral diseases, and more particularly on apoplexy. A memoir which he lately read on this subject before the Academy, and in which he not only impugned some of the prevailing notions as to the causes of this disease, but also attacked the principles of phrenology, gave rise to a very animated discussion among many of the most distinguished members of that learned assembly. We propose to give a brief account of the proceedings, as reported in the Archives Génerales.

M. Rochoux defines apoplexy to be "hæmorrhagie par rupture, suite d'une alteration du tissu de l'encephale." The hæmorrhage, he believes to be almost invariably preceded by a diseased state of some portion of the cerebral substance, and to be very rarely or never caused by the mere impulsion of the blood against the walls of the blood-vessels, whether the heart be hypertrophied or not, provided the substance of the brain be healthy. If, says he, the rupture of the vessels was determined by the action of the heart, we could not well explain how the cicatrization of the "foyers hæmorrhagiques" could ever take place; seeing that the cause, which primarily induced the mischief, was still in operation. The examination, too, of these "foyers" when recent, leads us to the same view of the question. In those frightful cases of apoplexy, in which the patient is stricken dead almost at once (the "apoplexie foudroyante" of the French authors), we find that the cerebral substance, which is in immediate contact with the effused blood, "est herissé de lambeaux rouge-brun, flottant dans le foyer, comme une sorte de gazon," and that it is impregnated all round, to the depth of from half a line to two or three lines, with bloody dots; these dots or specks becoming less and less distinct from the centre outwardly. This dotted stratum is succeeded or enveloped by one which is of a yellowish hue. Both these strata of cerebral matter are always, according to M. R——, decidedly softened in texture. M. Rochoux accounts for the occasional absence of any cephalic annoyance before the apoplectic seizure, by the analogy of other important lesions of organs, which are detected only on post-mortem examination, no symptoms during life having indicated their existence. Apoplexy occurs quite as often when the circulation is calm and not excited, as when it is over-active and accelerated. During youth apoplexy is rare, and it becomes gradually more and more frequent in mature and in advanced age. Insane persons are frequently affected with cerebral congestion, but very rarely with genuine apoplexy.

M. Rochoux has compared the state of the heart in 30 apoplectic patients, with its state in 30 other patients, who had died of different diseases—the ages of the two sets of patients being nearly alike, from 60 to 70 years. In 24 of the first set of cases, the heart was found more or less hypertrophied; whereas, of the second set, it was similarly diseased in not fewer than 26. The influence of the heart, therefore, in predisposing to apoplexy is at least far from being very decidedly established.

M. Rochoux having thus impugned the etiological doctrines maintained by Broussais, Bouillaud, Hope, and others, regarding apoplexy, passed on to the consideration of the symptoms of the disease. He remarked that, whatever be the seat of the cerebral hæmorrhage, the whole, and not one part only, of the brain suffers, and that the general or constitutional symptoms are always the same in character.

Does not this well-ascertained fact invalidate, asks M. Rochoux, the truth of phrenology? Taken in connexion with the numerous inconsistencies which we constantly meet with in the doctrines of the advocates of this system, as well as the contradictions afforded by the examination of the heads of noted criminals—Fieschi, for example—we do not hesitate, says M. Rochoux, to assert that "la phrenologie est un des plus grands mecomptes de l'époque." This bold avowal appears to have fired the wrath of many of the academic members, and an animated discussion followed.

We shall first attend to the answers of M. Rochoux's opponents, in reference to the alleged influence of the heart in inducing apoplexy. M. Piorry, whose name is creditably known to the profession by his auscultatory researches, took the lead in reply. He endeavoured to shew that M. Rochoux had confined his attention too exclusively to the state of the left side of the heart in apoplexy, and that he had omitted to take any account of the retardation and stasis of the blood in the encephalon, induced by affections of the right auricle and ventricle. When both sides of the heart are simultaneously diseased, the injurious effects on the brain are greatly magnified; for while the blood is propelled into the cerebral arteries with unusual violence, in consequence of the hypertrophied left ventricle, its return through the veins is impeded by the gorged state of the right side of the heart. The statistical statement adduced by M. Rochoux loses, therefore, its force, by the defect of all information as to the state of the right side cavities in the 30 cases of fatal apoplexy. The arguments urged by M. Piorry in favour of the alleged influence of diseased heart, as a predisposing cause of apoplexy, are these: the attack is usually sudden, and not announced by premonitory symptoms—the patients are commonly fat, plethoric, and short-necked—and the most frequent occasional causes are strong bodily exertions or efforts.

That ramollissement of the cerebral substance should generally precede an apoplectic hæmorrhage is rather improbable, seeing that hæmorrhages often occur in other organs, unattended, as far as we know, with softening of their texture. That the parietes of the cerebral bloodvessels themselves are, in apoplexy, very commonly diseased—more friable or lacerable than in health, in consequence of calcareous depositions in their coats—is admitted by all pathologists; but certainly this state of the vessels is not always associated with ramollissement of the adjacent cerebral substance. The circumstance, too, of the sanguineous effusion being not unfrequently on the surface, and not within the substance of the brain, may be adduced in support of this view of the subject—that softening of any part of the brain is by no means a necessary accompaniment or precursor of apoplectic hæmorrhage. That this morbid state is occasionally present in cases of apoplexy, M. Piorry is quite ready to admit; but he is of opinion that, in by far the larger proportion of cases, the cerebral hæmorrhage is associated with some abnormal state of the heart and of the lungs.

M. Rochoux, in answer to these objections urged by M. Piorry, asserted that he had never met with a single case of a "foyer apoplectique," unaccompanied with ramollissement of the surrounding cerebral substance, and challenged his opponents to shew him such a case. M. Bouillaud, on the other hand, contended (such is the accuracy of medical observation, and such the value of medical testimony!—Proh pudor!!) that he had never seen an example of genuine ramollissement of any part of the brain associated with cerebral hæmorrhage. Of 54 cases of hypertrophy of the left ventricle, cerebral hæmorrhage existed in six, and ramollissement in five.

M. Rochoux answered that he had never denied altogether the influence of the state of the heart and arteries in producing apoplexy, and that he had merely asserted, that such influence is, on the whole, very inconsiderable, and not easily to be appreciated.

As to the ossification of the cerebral arteries, we should remember that this morbid state has never been detected in any vessels beyond the pia mater, and which are imbedded in the substance of the brain.

M. Louis stated that he belonged to neither party of disputants, and that, while he did not admit the theory of M. Rochoux that cerebral ramollissement always precedes apoplectic hæmorrhage, he was very far from assenting to the doctrine of his opponents, that the disease in question is very generally associated with hypertrophy of the heart. The two morbid states of the cerebrum, viz. ramollissement, and hæmorrhage on its surface, or within its substance, are by no means connected with each other in the relation of cause and effect. They differ in many respects: for example, they differ in regard to their seat or locality, to their progress, and to their termination. The former affection is observed chiefly in the cortical matter of the brain, in the septum lucidum, in the centrum ovale of Vieussens, and in the fissure of Silvius: the latter is most frequent in the corpora striata.

Ramollissement occurs usually in several points at the same time;—hæmorrhage is almost always single and isolated. Ramollissement is very often complicated with other morbid states—apoplexy is rarely so. Lastly, in respect to the termination of ramollissement and of apoplexy, these two affections differ most materially. The former is perhaps quite irremediable: while the latter is not unfrequently cured. It is only in regard to the mode of their commencement (debut) that these two diseases occasionally resemble each other; for if the attack of apoplexy is in one-third or fourth of the cases sudden, that of ramollissement is occasionally not less rapid, especially when the corpus striatum is the part affected.

Such are the opinions of M. Louis on the connexion of apoplexy with ramollissement of the cerebral substance. With respect to the influence of the heart in inducing the former disease, he quite agrees with the sentiments professed by M. Rochoux. Of 45 fatal cases of organic disease of the heart examined at La Charité, not one had exhibited any symptoms of apoplexy during life, nor any traces of cerebral hæmorrhage after death, and the researches of M. Louis at La Pitié have presented the same results. The cerebral congestions, which are most apt to supervene in cases of heart-disease, are serous and not sanguineous.

M. Rochoux replied to these observations of M. Louis, by stating that there are two sorts of ramollissement of the brain, very different the one from the other. The first is the consequence of an inflammatory action in the part affected, is invariably fatal, and may be recognized on dissection by the soft diffluent state of the cerebral matter, when a small stream of water is directed upon it; whereas, in the hæmorrhagic ramollissement, the cerebral substance is, as it were, reticulated, and is not so diffluent under a stream of water.

M. Bricheteau could not agree with M. Louis, that the action of the heart has little influence in producing apoplexy. He has published the histories of 35 cases of the disease, and in most of these there existed an hypertrophied state of the heart. As to the objection of M. Rochoux, that hypertrophy of the heart was very rarely present in the cases published by him, it is worthy of notice that all his patients were old men, who are seldom the subjects of hypertrophy of the heart. On the contrary, M.M. Bricheteau's patients were under sixty years of age, and many of the cases recorded by M. Bouillaud occurred in middle aged persons. Perhaps therefore the difference in point of age of the patients may account in some measure for the difference in the results. M. Piorry detailed the case of a young boy, affected with hypertrophy of the heart, who had experienced repeated cerebral attacks, and had within the last few weeks been seized with hemiplegia. Here closed the discussion on the causes of apoplexy; and the other topic of debate—phrenology—was introduced by M. Bouillaud. He took a rapid view of the rise and diffusion of this new system of mental philosophy, and shewed very ably that its leading principles had been recognized by many of the wisest physiologists long prior to the time of Dr. Gall. M. B. did not hesitate to avow, that while he believed in the general axioms of the science, he was very far from yielding an assent to all its details, as expounded in any publication on the subject.* For example, he did not think that the feeling of sexual desire, or, as it is denominated, amativeness, has any connexion with the cerebellum,—the function of which is in his opinion to preside over and regulate the equilibrium of the muscular system in walking and in other movements of the body.

M. Rochoux was delighted to hear his honorable confrere make so liberal an avowal of his sentiments, and he was anxious thus publicly to call attention to the concessions now elicited; for the main scope of his attack on phrenology was directed against that mania for the localisation of mental faculties, which induced Dr. Gall in the first instance to portion out the brain into 27 different sections, and his followers to add nine additional sections to the number.

M. Broussais "prenait la parole." He was not prepared to defend all the opinions either of Dr. Gall, or Spurzheim, or of any other phrenologist. Many of the details of authors on this, and indeed on every other branch of science, are necessarily imperfect and inaccurate; but such an objection cannot invalidate its leading principles and conclusions. These have been deduced from a patient examination of facts, which no more reasoning can gainsay, and which most satisfactorily establish this important truth, that certain mental developments are always associated with certain cerebral formations. This "empiric" fact is the foundation of all phrenological reasonings, and notwithstanding the indiscreet and ignorant haste of many disciples of the science, its essential doctrines are based upon the most incontestable observations.

M. Rochoux has rather contemptuously asked us to shew him those various organs of the brain, which exercise different functions, and which are alleged by us to preside over different powers of the mind. No discreet phrenologist has ever professed to point out the different portions of the brain, as

* In his recent work on medical philosophy, M. B. has expressed sentitiments to the same effect. His words are—"Sans doute, il reste infiniment à faire pour la determination precise du siege des facultés intellectuelles et morales, et de leurs instruments; sans doute, cette science naissante est enveloppée encore d'obscurités, de doutes, d'incertitudes. Mais la base meme de la doctrine repose sur une si nombreuse collection de faits et d'observations, qu'elle est inebranlable." He then alludes to the incredulity of M. Magendie, who ranks phrenology with necromancy, alchemy and astrology, and has stated his opinion in this sentence:—"Les efforts de cette pseudo-science reduisent à des assertions, qui ne soutiennent pas un instant l'examen." M. Bouillaud in reply says—"En vain Cuvier et Napoleon lui-meme s'opposerent à la doctrine de Gall. Elle triompha de leur resistance, et par une vengeance digne d'elle, elle se sert des tetes de ces deux grands hommes pour appuyer ses principes. Apres une telle victoire, quels ennemis la phrenologie bien comprise aurait elle desormais à redouter?"

if they were separated and distinct organs. Like the faculties of the mind itself, they are intimately and indissolubly connected together, and it is impossible to ascertain the exact boundaries of each division or cerebral organ. Of late years, little has been said by the opponents of phrenology of that argument, drawn from the want of perfect correspondence between the inner and outer surfaces of the cranium; an argument which was so boastfully dwelt upon twelve or twenty years ago. The phrenologist does not require the measurement to a line, either in point of extent or of prominence, of organs, before he can estimate the prevailing character of any individual. The abandonment of this weak objection shews that a very great change has taken place in the sentiments of those who are still hostile to phrenology. It has been said by some that comparative anatomy is opposed to our science; for example, that the brain of many of the lower animals very much resembles that of a human being, and yet their mental endowments are few and feeble. This argument when examined lends but little support to the opponents of phrenology. It is very doubtful that man possesses a much greater number of mental feelings and faculties than many of the lower animals. In them they exist probably " en germe, en esquisse." No one will deny that they are endowed with the feelings of love for their offspring, and of attachment to their abodes; and we think that it is equally true that they possess a memory for persons, things and places, as well as a perception for sounds and distances. Who will deny that pride, or some feeling close akin to it, is felt by the cock surrounded by his feathered dames, by the pigeon strutting about with puffed-out neck, and the pea-fowl displaying his gorgeous train. Is there not something almost human in these exhibitions of self-esteem and love of admiration?—Glimmerings of ideality, (?) of judgment, of comparison, of veneration, of hope, &c. are often observed in some animals, and most conspicuously in those which approach nearest in organization to the human development. Does not the lively joy of the dog, when unkennelled for the chase, betoken something of the workings of an imagination, which recals to him "des plaisirs qu'il a deja goutés'? and are we wrong in saying that this most faithful animal has a feeling of veneration (we do not say, theosophy) for his master?

We have heard lately a great deal of certain direct proofs—as they have been called—drawn from the measurement of the heads of notorious criminals, against the truth of phrenology.

The language which has been used by our opponents must satisfy every candid enquirer that they have not at all understood the doctrines, which they were assailing. It is rare that a murder is committed from mere " amour du meurtré," or from the direct impulse of the feeling of destructiveness. Other baneful passions too frequently urge on to the commission of this most dreadful of sins. Jealousy, revenge, avarice —these are often the prompters of the murderous deed. Again it has been urged against phrenology that very few scientific men, distinguished for their attainments in mental or in physical philosophy, have announced their assent to the truth of its doctrines. This indeed is a most feeble objection. In all ages, the "savans" have been the most opposed to the diffusion of "deconvertes nouvelles." The dread of ridicule, envy, the reluctance to be taught any subject by younger and less lettered men, and a host of other feelings—"voila tres souvent les mobiles de cette opposition."

M. Broussais is ready to admit that phrenology is far from being yet complete as a phrenological or descriptive system of all the cerebral functions, and that the localisation of certain faculties is still imperfect. What remains to be done may be easily accomplished by the careful and repeated examination of the heads of those, who are conspicuously distinguished for any particular mental endowment. There may be difference of opinion as to the exact limitation of certain organs; but such a discrepancy on smaller matters affords no solid objection to the fundamental positions of the science.

That education and the exercise of particular faculties will, at least in youth, induce a larger development of the corresponding cerebral organs, than if no such education or exercise had been undergone, M. Broussais has repeatedly assured himself by minute observation; but we are to remember that it is only "quand il n'y a pas de predominance speciale, que le cerveau va comme il est *dressé*."

The next speaker was M. Adelon, one of the most scientific members of the academy. He observed that M. Rochoux was not quite correct in stating that in apoplexy, whatever may be its seat, the "ensemble" of the brain suffers, and that the general symptoms are the same in all cases. Cerebellar apoplexy has been very frequently diagnosticated by the presence of involuntary erections of the penis.*

With respect to the labours of the phrenologists, M. Adelon very justly remarked that surely they are quite as deserving of scientific examination as the attempts of anatomists to measure the facial angle and the area of the cranium in animals—attempts which have engaged the attention of such men as Camper, Daubenton, Cuvier, and Soemmering. All these enquiries rest on the same foundation, and have the same object in view—to discover, by the formation of the (anterior part) head, the amount of intelligence possessed. Moreover every series of experiments on the brain and nerves performed, within the last fifty years, has tended "a pluraliser le systeme nerveux." The divisions of Bichat, the researches of Charles Bell, Magendie, Flourens, Foville, &c. all tend to this result, and the conclusions, which these eminent physiologists have come to, have met with very general assent. If phrenology has more lofty pretensions, aiming, as it does, to penetrate into the depths of mental philosophy, it encounters, we must acknowledge, difficulties numerous, great, and perhaps insurmountable. But be it remembered that it professes to have been built, and still to advance on the results of cautious, minute and repeated observation; and if so, although the subject may be still encompassed with difficulties, it does not become the man of science to reject or despise its labours. The "point culminant" in phrenology is, first to determine the number of faculties, and then to ascertain the precise localisations of these faculties or endowments. *Perhaps we shall never arrive at exact and indisputable truth on such questions;* and "c'est là la veritable et la seule objection rigoureuse qu'on ait faite." In conclusion, M. Adelon expressed his opinion that it was rash and unphilosophical in any one to profess his contempt of phrenology, until he has minutely and most attentively studied the subject. Few medical men have leisure enough to devote to such difficult enquiries as those involved in phrenology.

M. Amussat professed himself favourable on the whole to the conclusions of phrenology. He denied the assertion of M. Rochoux, that the history of apoplectic symptoms affords any just objection. Many cases of the disease seem to confirm the doctrines of M. Gall. With respect to the alleged inconsistency of the cranial developments in Fieschi and other notorious assassins with their well-known characters, M. Amussat was of opinion that many incorrect statements had been made by the friends, as well as by the foes, of phrenology. It has been asserted that the head of Fieschi is

---

* M. Recamier, well known as a most experienced and able physician, stated, as the result of his experience, that, when the cerebral hæmorrhage was on the anterior lobes, the organs of speech were very generally paralysed.—M. Bouillaud confirmed the truth of this remark, and observed that the loss of speech was in some cases of apoplexy unaccompanied with any other mental infirmity. It is necessary not to confound the mere paralysis of the tongue with the loss of the faculty of speech. The movements of the tongue may be quite free, and yet the patient cannot articulate a word. The cause of this special aphonia is unquestionably some lesion of the anterior cerebral lobes.

not to be distinguished from that of an "honnete homme." Now in truth it is (shewing the mould) the head of a miserable wretch.* It is of small dimensions. The organs of pride and firmness—the most prominent and "motivant" features in Fieschi's character—however are of considerable size. The lateral depressions in a murderer's head may at first sight surprise us; but, be it remembered, that Gall has never said that a person may not be an assassin "sans l'organe du meurtre." The conformation of the head of Fieschi accounts for, if not his last and most atrocious crime, the profligate dispositions of his general character. His destructive propensities were by no means so conspicuous as his inordinate and unprincipled love of notoriety. It did not appear that he had been urged on to his villainous acts by revenge or bloodthirstiness. He had no cause of resentment against the king or any of his attendants; but leading for a length of time an idle, unoccupied and unprincipled life, his heart had been open to some vague, and almost undefinable Satanic whisperings of personal distinction.*

MM. Maingault and Capuron—the former a most meritorious surgeon, the latter a distinguished physician-accoucheur—spoke in favour of phrenology. One remark which M. Capuron made deserves to be noticed. "I cannot," said he, "believe that the same organ is the seat of the reasoning principle which controls the passions, and of these very passions which so often overthrow the reason."—*Arch. Gener.*

M. Ferrus, in the introductory lecture to his course of clinical instruction† on mental diseases, at the great lunatic establishment of the Bicetre, thus expresses his opinion of the value of phrenology:—

"The method of studying and treating insanity recommended by MM. Gall

---

* The tone in which many of the French journalists have spoken of this and other atrocious assassins is, we fear, sadly expressive of the false and most dangerous sentimentality, which has so often misled the minds of their countrymen. For example, in a late number of the Gazette Medicale de Paris, we meet with the following fanfaronade: "Fieschi se pose avec ostentation sur le terrible piedestal qu'il s'est elevé, et semble dire aux hommes epouvantés qui le regardent: 'qui d'entre vous en eût fait autant?' Que sa tete tombe, c'est bien; mais qu'on lui rende cette justice qu'il a bravement conduit, et executé son enterprise."

Another medical periodical, Lançette Française, after describing the characters of Lacenaire and Avril, (who, it will be remembered, were condemned to death about a twelvemonth ago in Paris,) and shewing that they, at least Lacenaire, had frequently committed murder from the basest and most villainous motives, favours us with the following precious morsel of jurisprudential reasoning. "Nous le demandons, á quoi sert la guillotine pour des hommes de la trempe de Lacenaire? A quoi sert il pour les hommes bruts et courageux comme Avril? A rien, absolument à rien; car, comme le dit Lacenaire, tout assassin est courageux. Reste alors le hideux du supplice dans toute sa nudité."

* We fear that there are many Fieschis and Alibeaus in the inhabitants of the French metropolis at the present moment. We cannot well appreciate—fortunately from want of examples—such characters in this more phlegmatic, calculating, and selfish country of ours. There are villains enough, alas! among our population, and crimes of almost every dye; but certainly there are few instances of "political assassination."—*Rev.*

† The example of M. Ferrus deserves to be imitated in this country. Insanity, like every other disease, can be advantageously studied only at the bedside of the sick. Hitherto all our public lunatic establishments are no better than so many "musea clausa." Now that medical jurisprudence is more attended to, we may hope that the study of mental disorders will occupy a prominent place in medical instruction.

and Spurzheim appears to us to be by far more philosophical than any other, and, if pursued with discretion, likely to lead to most beneficial consequences;" and a higher authority, M. Andral, in his Lectures on the Nervous System, has used these words :—

" La doctrine phrenologique contient de grandes choses, de belles acquisitions pour la science; mais malheureusement aussi elle renferme des niaiseries, qu'on nous passe expression. Ils ont donc tort ceux-la qui traitent Gall sans reserve, et affectent pour lui un superbe mépris; de meme qu'ils tombent dans un autre défaut, ceux qui adoptent ses opinions d'une manière trop exclusive."

---

### Illustrations of various Lesions &c. of the Nervous System.

We have associated in this article a number of detached notices, which, when standing detached and insulated, could only have excited our passing curiosity, but when linked together, may probably suggest some useful hints in the study of the Neuroses. The first case recorded exhibits a very striking example of one of the rare forms of that Proteian disease, hysteria—the stoppage of the urinary discharge from the bladder, and the vicarious secretion from the surface of the body, will attract particular notice.

The case of catalepsy, although one of short duration, was very characteristically defined: the pathognomonic sign of the disease—the limbs and body retaining whatever position may be given to them—was well marked. Baron Larrey's case of traumatic epilepsy, of 33 years' standing, and connected apparently with irritation from local disease seeing that the convulsions ceased on the removal of a diseased portion of cranium, is highly interesting and instructive. With respect to the treatment of idiopathic epilepsy by the nitrate of silver, or by other metallic salts, we may remark that it will succeed in some cases; but the experienced practitioner is well aware that this malady is too often the mere outward symptom of cerebral disease, to warrant great reliance on any particular medicine. Setons or issues in the neck should be generally employed at the same time, along with the use of metallic or of other tonics.

The cases of neuralgia, and more especially the first one, will be read with great interest. The severity and extreme obstinacy of the symptoms, the irregularity of the attacks, the almost total inefficacy of every kind of treatment, and eventually the happy recovery in a manner quite unexpected—such are often the features of this imperfectly understood disease. On the whole, internal remedies are not of much use; or at least they fail so often, that we can never place much reliance on them. Provided the constitutional health be good, more benefit will be derived from local than from general treatment. The veratrine and aconitine ointments will sometimes succeed when all ordinary means fail; but, as a matter of course, many cases will resist them. We observe reported by Mr. Skey in a late number of our contemporary, the Medical Gazette, two or three well-marked cases of severe neuralgia, in which decided benefit was derived from the aconitine ointment.

Strychnine, also, one of the most powerful alkaloids, may sometimes succeed; it may be used internally and endermically at the same time. We have adduced a few cases to illustrate its great utility in some nervous affections of the bladder, and especially in incontinence of urine, when depending on an over-excitable, or on an enfeebled state of the sphincter vesicæ.

We have appended a few observations on the medical uses of electricity and galvanism; and, lastly, we have extracted from one of the German periodicals a short account of the appearances found in the bodies of some animals, which had been killed by lightning.

### *Hysteria followed by Ischuria—Vicarious Discharge of Urine from the Mammæ.*

A young woman, 24 years of age, of a highly irritable temperament, and who had frequently suffered from violent convulsive attacks, was seized with a complete suppression of urine. For some days previously, she had felt a sense of painful contraction in the loins, over the kidneys, and along the lines of the ureters, extending occasionally down to the seat of the bladder. The abdomen was tympanitic and very tender, the bowels obstinately confined, the stomach irritable, and the intellect was wavering. There was a frequent desire to void urine; but the catheter brought away only a very small quantity of limpid inodorous water. The vomitings and other symptoms gradually increased in severity, and the suppression of the urine had continued for fifteen days, when the patient began to complain of acute pain in the axillæ and in both mammæ, which soon became swollen and tender. A limpid fluid, having a very distinctly urinous smell, oozed out from both nipples, with great relief to all the symptoms. This oozing continued for two days, after which it ceased; and, strange to say, it then took place from the umbilicus, and from the skin of both legs. The surface, from which this oozing took place, was bedewed with drops of a clear fluid, while all the rest of the skin was quite dry, and free from any moisture.

The discharge from the skin of the legs soon ceased, but the mammæ and umbilicus were still alternately affected with this extraordinary secretion—the former more frequently than the latter. After continuing for some time, the discharge ceased during several days (still no urine had been voided from the bladder), the abdomen began again to swell, and all the former spasmodic symptoms threatened to return, when all of a sudden, after a fit of the most agonizing sufferings, a fulness over the region of the bladder appeared, and there flowed from the urethra upwards of seven pints of deep, strong-smelling urine. Twelve hours afterwaids, she again passed upwards of four pints. From this date she remained free from any urinary irregularity; but six weeks afterwards, more than eight ounces of blood flowed from the mammæ. The catamenia had been irregular for a length of time. This sanguineous discharge returned at intervals; and sometimes it took place from the navel. The health of the patient continued to be much disordered for more than a twelvemonth, and when the above account was written, it appears that she was still suffering from dysmenorrhœa, attacks of spasm, and symptoms of vermination. On one occasion, she had voided a piece of a tænia cucurbitina. —*Caspar's Wochenschrift, April,* 1836.

*Case of Catalepsy—well-marked Symptoms—speedy Recovery.*

A middle-aged woman, of an active constitution, and who had long enjoyed good health, was brought into the Hôpital de la Pitié on the 15th of July, under the following circumstances. It appears that she had been engaged on the preceding day as a labourer in the hayfields, and that, feeling a troublesome headache, she had sat down on the ground, and had there fallen fast asleep. She had continued in this state during the whole of the night, and was found next morning by a countryman. She was immediately conveyed to the hospital, and M. Barth drew up an accurate report of the case. She was still in a deep sleep, utterly insensible to all ordinary impressions, and even to pinching and pricking of the skin. The breathing and pulse were natural. All the joints of the limbs were pliant, and easily flexible; and it was found that the legs and the arms remained steadfastly in the position in which they were placed. This singular character of the disease was most conspicuous, when the body was raised up on the bed, and the arms and legs were lifted up in the air at the same time. There she sat for some time, resting only on the ischia, in the strangest and most difficult position, her arms being stretched out, and her heels nearly as high as her head: by degrees the body sunk back, by gentle oscillations, on the bed.

Two sinapisms were applied on the inside of the legs. She soon began to be annoyed with their irritation, and to complain of the pain. The somnolence gradually abated, and towards evening she awoke, quite unconscious of what had passed, and surprised to find herself in a hospital. Next day she was quite well.—*Journ. Hebdom.*

*Epilepsy of* 33 *Years' Standing, cured by the Removal of a Carious Portion of the Cranium.*

One of the inmates of the Hôpital des Invalides, 63 years of age, had been wounded at the battle of Marengo with a piece of a small shell (obus) in the forehead. He was immediately trepanned, and a portion of the bone extracted. The symptoms of commotion and of compression were succeeded by those of encephalitis. When these subsided, he was found to be paralytic on the right side, and to have lost completely "la memoire des choses." The wound never fairly healed, but remained fistulous; and scarcely a day passed over without an attack of epileptic convulsions. For the last 33 years, he had been in this deplorable condition, subjected to daily fits, totally deprived of his memory of things, and having a fistulous sore on his forehead. M. Larrey having detected with a probe a loose portion of bone, divided the teguments, and extracted it with a forceps. The wound and sore speedily healed up, the attacks of epilepsy have ceased to return, and his locomotive and mental faculties have become considerably improved. We are informed, however, that his memory of numbers and of names is still very imperfect and wavering.

The reporter of the preceding case alludes to the confirmation which its history gives to phrenological science, and invites the disciples of Gall to study "la precieuse collection vivante" of heads, in the "asyle de l'ancienne valeur de nos armées."—*Lançette Française.*

*Epilepsy cured with the Nitrate of Silver.*

Two successful cases are reported in the March and April Numbers of the Italian Journal, Il filiatre Sebezio. In the first, which occurred in a plethoric man, 29 years of age, the disease had lasted for twelve years, and had gradually become more frequent and severe, so that the patient could not follow his occupation as a coach-driver. The medicine was taken in minute doses, 1-16th of a grain, at first; and by degrees the dose was increased to a full grain in 24 hours. The treatment was begun in June, and by October, the patient seemed to be quite well.

The subject of the second case was a youth, 20 years of age. In 1829 he was first seized with an epileptic fit, after the sudden stopping of an epistaxis. In 1831, Dr. Semenze visited him. As he was plethoric, and exhibited symptoms of congestion in the head, antiphlogistic remedies were used for two weeks, and then the use of the nitrate was commenced. It was given for ten days in the dose of a quarter of a grain; this was gradually raised to a grain and a half in the course of 24 hours; and this high dose was continued daily for a month. General bleeding, and the application of leeches to the anus had been used during the exhibition of the medicine. The cure was complete; and it seems to have been permanent, for there has been no return of the disease during the last three years.

*Case of protracted Neuralgia of the Spermatic Nerves, connected with a small Urinary Calculus, whose presence had not been suspected.*

A gentleman, 52 years of age, healthy and very robust, was suddenly seized with an excruciating pain in the left testicle. It extended up along the cord, and was accompanied with general spasm, repeated vomitings, and extreme anguish. This attack lasted for 15 hours, and then subsided gradually. On the following day he was easy, but on the next a similar attack was experienced. For some time the disease continued to return in this periodic manner, every third day. Quinine was, therefore, administered in large doses, and with a certain degree of benefit; for, at least, the intervals between the paroxysms were now longer, although their severity was almost as great as ever. Sometimes five, eight, or even fifteen days elapsed between the attacks, at the period when this gentleman went to Paris to consult M. Reveillé-Parise. The testicle and cord were most attentively examined; but no appearance or sign of any lesion could be discovered, and the general health of the patient was excellent. In spite of "bains, antispasmodiques et lavemens opiacés," the disease continued to return with the same intensity of suffering, and the attacks were as irregular as before. A rigid antiphlogistic regimen was tried, and this appeared to have for some time worked a decided benefit; but again the enemy re-appeared, and M. Marjolin was then consulted. This experienced surgeon could not detect any trace of morbid change in the affected part, and he agreed with M. Parise in regarding the case as one of neuralgia of the spermatic nerves, although in a thirty years' practice, he had never before met with a case of the sort.

The cord was repeatedly leeched; the loins were cupped; vapour-baths were tried; various antispasmodics and anodynes, internal as well as external, were exhibited—but all in vain. Once more the quinine was resorted to; but this medicine, as well as the subcarbonate of iron in large doses, the extract of aconite, the application of morphia, of cyanuret of potassium, &c. brought little or no relief to the patient's suffering. If one or two paroxysms were at any time relieved, the subsequent ones were more excruciating than ever. When the attack of pain had subsided, the patient felt as well as ever he did in his life, and all the functions of the body were vigorous and healthy. The urine was repeatedly examined, and it was found to be uniformly clear, and free from any sediment.

He left Paris, in despair of obtaining any relief to his malady. A short time after this period, he was very unexpectedly seized with a retention of urine. This was relieved by the catheter. A second seizure occurred a fortnight afterwards, and to the surprize both of patient and surgeon, he voided a small calculus, "dont la sortie determina aussitot et completement la cessation des douleurs."*

The preceding case is a most interesting and instructive one. If we suppose that the neuralgia was connected, from the beginning of the illness, with the presence of the calculus in the kidney or bladder, it is not easy to account for the intermittent character of the attack.—*Bulletin de Thérapeutique.*

### *Neuralgia.*

An obstinate case of neuralgia of the portio dura and frontal nerves, having resisted a host of remedies, external as well as internal, yielded at length to the application of a plaster, (composed of resinous plaster, of Venice turpentine, and of tartrate of antimony,) to the affected side of the face. It was kept on for several days. When it was removed, the skin was found to be covered with pustules and ulcers, but the neuralgic agony had quite ceased. The disease had not returned after the lapse of nine months.—*Medicinische Zeitung.*

We have known a plaster, composed of ceratum saponis, extract. belladonnæ, and acetas plumbi, act in the same

* Every surgeon knows that calculi will sometimes lodge in the bladder for a length of time, without occasioning much inconvenience. In a late Number of the Journal Hebdomadaire, Dr. Segalas reports a case, in which he found on dissection, in the bladder of an old gentleman, upwards of 300 calculi, all of which were composed of uric acid. The patient had not during life experienced any inconvenience, which could lead to the suspicion of such an extraordinary accumulation. Ten days before his death, he was seized with a retention of urine, which required the daily use of the catheter.

The bladder was found to be much hypertrophied; its mucous coat was reddened and inflamed. The kidneys were enlarged to twice their ordinary volume; the left one was softened in texture, and infiltrated with a purulent sanies.

manner and with like success as the one now mentioned.*—*Rev.*

*Curious Case of Metastasis of Neuralgia.*

An old man had suffered martyrdom from neuralgia of the left portio dura for 26 years. His agonies were sometimes quite dreadful; the slightest touch of the affected surface causing a violent spasm of the muscles of the face. The pain he compared to darts of lightning passing across from the ear to the nose. The general health had remained good during the whole time. Every remedial means had been ineffectually tried, and the only relief, which the patient could obtain, was from immense doses of laudanum taken every day. In 1831 he was suddenly seized with intestinal inflammation, which was attended with extreme sensitiveness of the abdomen, so that the gentlest touch brought on convulsions of the abdominal muscles: the neuralgia of the face quite ceased, and the cheek could be freely pressed and moved without any inconvenience. When the abdominal attack subsided, the neuralgic affection of the face returned, but certainly not with the same severity as before. Four days afterwards it again ceased, and the patient suffered from a burning pain in a circumscribed spot on the left hypogastric region. [The report stops here.]—*Hufeland's Jour. der Heilkunde.*

* One of the German journals gives a formula for the preparation of the balsamic plaster of Schauffans, (who received 30,000 dollars from the Russian Court, for disclosing the secret of its composition,) which had once an extensive repute for the cure of rheumatism, palsy, hemicrania, deafness, loss of vision, &c. The formula is the following:—

℞. Olei olivar, ℔iij.

Saponis Veneti, ℔j.

Pulv. cerussæ Venetæ, ℔j.

—— sulphur. hydr. rub. ℔j.

—— camphoræ, ℥iij.

—— castorei, ℥iss. Misce.

If the plaster thus prepared is not firm enough, a portion of wax should be added.

*Utility of Nux Vomica in Treatment of Incontinence of Urine, and Weakness of the Generative Organs.*

Incontinence of urine, whether it occurs in young children, or in old persons, is very generally dependent upon a relaxation of the sphincter vesicæ. The children, who are most liable to this annoying complaint, are of a weak lymphatic temperament, and usually exhibit the signs of a scrofulous and rachitic tendency. Laughing, coughing, &c. are often sufficient to cause the urine to escape. It is well known how apt these children are to urine in bed. Various remedies have been recommended by different practitioners. M. Dupuytren had great confidence in the daily use of the cold plunge bath. (This too was his favorite remedy against chorea.) MM. Baudelocque and Guersent also, two good authorities on the diseases of children, approve of cold bathing. The late Dr. Underwood recommended sea-bathing; and M. Lallemand of Montpelier informs us that no case of incontinence of urine in young persons has ever resisted the continued use of aromatic baths. These baths are prepared by infusing several handfuls of some of the labiate order of plants, called the "aromatic species," in boiling water, until it becomes of an agreeable coolness. Just before the child is put into the bath, a wine-glassful of brandy is to be mixed with it.

M. Baudelocque has lately tried at the Hôpital des Enfans the occasional introduction of a catheter into the bladder with good effect. Blisters and electricity also have been found of utility. The internal remedies, which have been most highly praised, are cantharides and nux vomica. Cantharides may be administered in the form of tincture—from five to twenty drops for a dose—or of the powder in pills. The formula used by M. Seiger is the following:—℞. Pulv. cantharid. gr. vj. Extract. boraginis, ʒij. Misce et divide in pil. xxiv.—one of these to be taken every night. More lately the extract of nux vomica has been strongly recommended by a number of physicians in France. A case was published by M. Mondiere in the

Archives Générales, which attests the great efficacy of this drug. A young female, 20 years of age, had been affected with nocturnal incontinence of urine, since she was six years old. The following prescription was written for her. ℞. Extract. nucis vomicæ, gr. viij. Oxydi ferri nigri, ʒj. M. et in pil. xxiv. divide. One pill was ordered to be taken three times daily. The incontinence had ceased before she had taken all the pills. The use of them however was continued for two or three weeks longer, to prevent any relapse of the complaint.

M. Lafaye has reported in the Journal de Med. de Bordeaux the case of an old man, who had been long distressed with incontinence of urine, and in whom a cure was effected in the course of seven weeks by the use of the extract, given in doses of from four to eight grains daily.

MM. Trousseau and Pidoux treated successfully in the Hôtel Dieu a case of paralysis of the bladder and of the rectum, which had been caused by an injury of the spine. These two physicians, in a well-written memoir on the medicinal virtues of the nux vomica published in the Journal des Connoissances Medico-Chirurgicales for May last, allude to its effects on the organs of generation, and report the case of a man who had been affected for a length of time with complete paraplegia, accompanied with general trembling: the thoracic and abdominal limbs, the bladder and the rectum, had quite lost their contractility, although the sensibility of these parts remained. From the commencement of the illness, the excitability of the generative organs had been destroyed. Under the continued use of the nux vomica, the paralysed parts recovered their motility, the tremors ceased, and the patient began to have erections of the penis.— These authors mention the particulars of another case somewhat similar, but not so severe, in a middle-aged man; and in him too the effects of the nux vomica on the penis were indubitable: "nous sommes parvenus à lui donner une virilité." The pharmaceutical preparation, which MM. Trousseau and Pidoux prefer, is the fresh alcoholic extract. The dose varies from one to fifteen grains per diem. The alcaloid —strychnine—is much more powerful. It is associated in the nut with another alcaloid, which has been called Brucine, and with an acid to which MM. Magendie and Pelletier gave the name of Igasuric. The same principles exist, but in different proportions, in the St. Ignatius bean.

### *Medical Uses of Electricity.*

There is an immense medico-electrical institution in Paris under the direction of M. Le Molt. From a report of the practice pursued there during the course of last year, we find that this most potent agent is still extensively used in the treatment of many diseases. Of late years electricity has been too much neglected by English practitioners. Like most other remedies, when first introduced, it was eulogised too highly; and hence, partly from its often failing to produce the expected effects, and partly from its application being not always of easy attainment, it has for some time fallen into unmerited disuse. There are four different forms in which electricity may be administered —frictions with an electrical brush; a mild and often very serviceable form in recent cases—the subtraction and insufflation, by means of, either aigrettes (bunches of feathers) of various sizes, or, what is still better, of the electrical brush—sparks, either communicated to or withdrawn from the part—and lastly, shocks or currents transmitted from one part of the body to another. It is well known that the last form is the most powerful of all. Galvanic electricity also was occasionally used with great success in some of the cases. The diseases, in which electricity has proved most useful at M. Le Molt's establishment, are various kinds of paralytic weakness, whether this proceeds from cerebral or spinal lesions, or from the poison of lead; chronic rheumatism and neuralgia; hysteria and hypochondriasis; atony of the urinary bladder; sterility, leucorrhœa, amenorrhœa, and weakness of the generative organs; deafness; and defects of vision.

All the cases of palsy, induced by the action of lead, were successfully treated. The mere electrical frictions, without having recourse to sparks or to shocks, were generally sufficient. In the palsies connected with cerebral or spinal lesion, the cases were fewer in number, and much more difficult of attainment. Great caution was necessary in administering the remedy in its powerful form over the head and upper portion of the spine. It is always proper to commence with the frictions and sparks, before having recourse to the giving of shocks.

Perhaps no set of cases are on the whole, more decidedly benefited by electricity than chronic rheumatism, complicated, as it often is, with "symptômes paralysiformes."

There are certain internal remedies, the use of which may, in most cases, be advantageously associated with electricity. Of these, strychnine is the most potent; and it is a curious circumstance, that this drug has the effect of inducing in palsied parts sensations, not unlike to those occasioned by electricity. When strychnine is applied to the excoriated surface of the eyelids or temples, the patient often perceives sparks issuing, as it were, from the eye itself. This is always a favourable symptom in treating amaurosis with strychnine. Frictions with a concentrated tincture of strychnine on paralytic parts have been occasionally used with benefit. Veratria is another of these remedies. Its power of causing a most troublesome tingling and pricking over the whole surface is certainly very strange, and appears to indicate that this alcaloid exerts a marked effect on the extremities of the nerves. We have not room at present for inserting more than one of the cases. It was an example of paralysis of the tongue, cured, or at least relieved, by galvanic electrity.

The patient, a man 45 years of age, had lost the power of speech, in consequence of an apoplectic attack, for 18 years, when he applied to Dr. Palaprat. An acupuncture-needle was inserted into the nape of the neck, in the direction of the basis of the brain. A communication was then established between the needle and the negative pole of a powerful Voltaic pile; and on the tongue was placed a small plate of platina, enveloped in linen which had been moistened in a saline solution, and this plate was put in communication with the positive pole of the pile. The patient experienced "commotions graduées," which gradually became so strong, as to make him be sensible of bright sparks before his eyes, an insufferable metallic taste, and violent contractions in the tongue and stomach. The dumb man, uttering an unusual cry, started forwards, articulating these words: "Je parle, monsieur—je parle, merci." After submitting to five other séances, he was able to pronounce every letter, and to repeat the words which were spoken to him. By continuing a proper discipline of utterance for several months, this patient was able "repeter plusieurs phrases d'une manière très intelligible, mais qui tendait toujours à devenir confusé."—*Journal des Connoissances Medicales.*

### *Post-mortem Appearances in Cattle killed by Lightning.*

A peasant, with a herd of 27 cattle, had taken shelter under a tree during a thunderstorm. He fortunately escaped, but every one of his beasts was killed by the lightning. On examining them, numerous dark zig-zag lines or streaks, produced by the scorching of the hair, were observed on their left sides, extending from the neck and shoulders towards the flanks and udders. These streaks varied in breadth, from a quarter of an inch to two and three inches.

A high degree of putrefaction had already (21 hours after the accident) taken place in all the bodies: this had been favoured by the hot weather. The stomach and intestines were enormously distended with gas, and a dark bloody-looking, offensive fluid oozed from the nostrils, muzzle, anus, &c. The mucous membranes, at these different openings, had a blue or blackish hue. The cellular membrane under the skin, in the course of the streaks, was found to be ecchymosed, and the veins in every part of the body were gorged with a very black and thin blood. All the viscera were more or less congested. The spleen was quite flaccid, and of a pappy consistence. The blood in it and in the venæ cavæ had quite the appearance of tar.

In one beast, the right half of the tongue was denuded of its epithelium, the velum palati was divided, the epiglottis destroyed, and the trachea cleft along its whole length, as if it had been divided with a knife : the trachea and likewise the bronchi were filled with clots of black blood. No lesions, such as now described, were found in any other of the cattle. In all, the right cavities of the heart were gorged with tarry-looking blood. The brain and other parts of the nervous system did not exhibit any abnormal appearance. —*Medicinische Zeitung, No.* 10.

---

### Poisoning from Colchicum.

Mad. de B. 25 years of age, swallowed a large wine-glassful of the tincture of the bulbs of colchicum, with the view of destroying herself. She was immediately seized with most severe pain in the epigastrium. Two pints of milk in about half an hour after the act had been committed, and subsequently two grains of emetic tartar were given. Copious vomiting of a brownish-coloured fluid ensued. M. Caffe did not see the patient till nearly six hours after the accident. He found her cold and very pale, complaining of tenderness in the epigastric region, a sense of constriction around the chest, and of great dyspnœa. There was no stiffness, nor any convulsive movements of the limbs ;* the lips were of a purple hue, the pupils were not dilated, nor was the vision affected ; the tongue was pale and cold to the touch; there was an almost constant vomiting, but no purging; the pulse was very slow and thread-like, and the patient, who was quite sensible, was tortured with a burning thirst, and every now and then she uttered the most distressing moans. M. Caffe administered iced aërated lemonade, and ordered sinapisms to be applied to the feet, and embrocations to the limbs and abdomen.

On the following morning, there was complete prostration of all the vital powers ; the retching still continued, and the cramps in the feet, although not so severe, were still present. Sinapisms were applied to the thighs, and a few leeches to the epigastrium. The patient became gradually weaker, and death took place in the afternoon, 22 hours after the poison had been swallowed.

The body was examined 70 hours after death. Putrefaction had already commenced. Most of the parenchymatous viscera were congested with dark-coloured and fluid blood. The stomach and intestines were removed and set apart, for the purpose of analysing their contents. On the following day they were examined; but unfortunately putrefaction had already advanced so far, as to have altered considerably the appearance of every part. The mucous membrane of the intestinal canal, in different places, was of a wine-red colour; but no dependence could be placed on the results of such an examination. Equally unsatisfactory was the chemical analysis of the contents of the bowels.

Strange to record, the sister of this patient, a girl of about twenty years of age, made a similar attempt on her life, some months afterwards, by drinking between four and five ounces of a weak vinous tincture of colchicum. The symptoms were nearly the same as in the preceding case; violent pains in the epigastrium, constant vomiting, without however any diarrhœa, a sense of constriction around the chest, extreme dyspnœa, gradually-increasing coldness of the whole surface of the body, and absence of convulsions, excepting in both feet. The pulse was small and very

* It is afterwards stated that the soles of both feet were affected with most painful cramps.

feeble, the pupils were not affected, and the mental powers remained to the last undisturbed. Milk, iced drink, emollient enemata and blisters to the extremities were the means used, but without avail. The patient died on the following day, 28 hours after the swallowing of the poison.

The body was examined 43 hours after death. The stomach, much distended with gas, contained several spoonfuls of a turbid, inodorous fluid; its mucous membrane was quite exempt from any appearance of inflammation or unusual vascular congestion, but its texture was much softer than usual, so that it could be easily detached from the submucous tissue in the form of "un detritus pultacé." Throughout the whole tract of the intestines, the muciparous glands were observed to be very prominent and distinct, and in the lower third of the ileum, the areæ of the conglomerate glands were of a livid colour. The other viscera did not exhibit any obviously abnormal appearances.

*Observations.* The symptoms in these two cases were quite alike, and sufficiently prove that the action of colchicum in large doses is on the nervous system, and may prove fatal, without inducing any excessive evacuations. The circumstance of the cramps being confined to the feet in both cases deserves to be attended to. There is no mention of this symptom having been observed in the case recorded several years ago in the Edinb. Med. and Surg. Journal, of a man who died on the 3d day after swallowing an ounce and a half of tincture of colchicum. In this case, there was also great diarrhœa, and delirium came on before death.

Bernt has noticed (vide Christison on Poisons) the cases of two children, who were poisoned by a handful of colchicum seeds, and who died in a day, affected with violent vomiting and purging. In the bodies of these children there was considerable redness of the stomach and small intestines; in the case mentioned in the Edinburgh Journal there was no morbid appearance at all to be found.

It is generally believed that colchicum owes its active qualities to the presence of veratrine, and as Magendie has shewn that this alkaloid, when introduced into the stomach, induces tetanic convulsions, we might have expected to meet with them in poisoning from colchicum; yet these symptoms were absent in the two preceding cases. —*Archives Générales.*

In reading over some of the late numbers of Hufeland's Journal we have met with the following truly-German account of the effects of an overdose of the tincture of colchicum. We give the report, nearly as it stands in the Journal.

That the colchicum, first recommended and employed in England against the gout, should have met with a less favorable reception in Germany, we need not perhaps be much surprised, seeing that the disease is greatly less frequent with us than among the English. Climate, mode of life, and other influential agencies, may very possibly have contributed to have given to *our* enquiries a different colouring, and to *our* observations very different results, from those which have been noticed by foreigners. The effects of colchicum are, according to the unanimous reports of all witnesses, purely and altogether physical. It appears to exert a specific effect on the nerves of reproduction, on the ganglionic system, on the skin, kidneys, the mucous membranes, and in an especial degree on the function of secretion (und uberhaupt auf das secretions-vermogen.) It is, in all probability, owing to its power of causing an increased flow of the intestinal secretions, and of removing venous plethora, that colchicum is so useful a remedy in gout.

An old gentleman, having taken a large dose of colchicum, was attacked soon afterwards with headache and a peculiar irritation of the cerebral nerves. His *corporeal vision* became more acute, and his *intellectual* vision so much more obtuse, that he could not now explain the words, which he read with his sharpened sense.(!) The movements of the tongue were difficult and irregular. The stomach and bowels were not affected.

(Voila! a sample of some contents of the German journals.—*Rev.*)

